# Supplementary material for: Mutations in APOPT1, Encoding a Mitochondrial Protein, Cause Cavitating Leukoencephalopathy with Cytochrome c Oxidase Deficiency
Source: Am J Hum Genet. 2014 Sep 4;95(3):315–25. doi: 10.1016/j.ajhg.2014.08.003 (PMC4157140; doi:10.1016/j.ajhg.2014.08.003)
Supplement: Document S1. Figures S1–S8 and Table S1 [file mmc1.pdf]

The American Journal of Human Genetics, Volume *95*

Supplemental Data

Mutations in *APOPT1*, Encoding a Mitochondrial Protein, Cause Cavitating Leukoencephalopathy with Cytochrome *c* Oxidase Deficiency

Laura Melchionda, Tobias B. Haack, Steven Hardy, Truus E.M. Abbink, Erika Fernandez-Vizarra, Eleonora Lamantea, Silvia Marchet, Lucia Morandi, Maurizio Moggio, Rosalba Carrozzo, Alessandra Torraco, Daria Diodato, Tim M. Strom, Thomas Meitinger, Pinar Tekturk, Zuhal Yapici, Fathiya Al-Murshedi, René Stevens, Richard J. Rodenburg, Costanza Lamperti, Anna Ardissone, Isabella Moroni, Graziella Uziel, Holger Prokisch, Robert W. Taylor, Enrico Bertini, Marjo S. van der Knaap, Daniele Ghezzi, and Massimo Zeviani

Supplemental Data

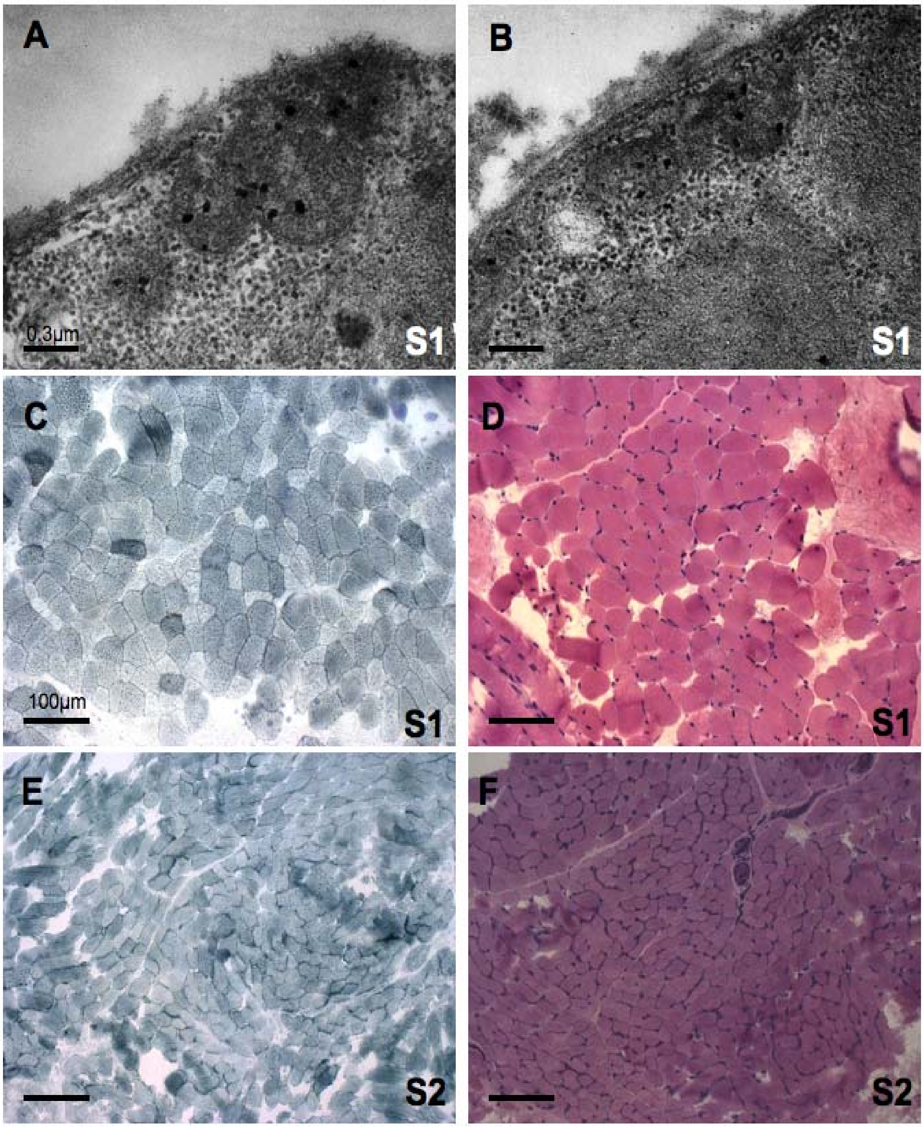

Figure S1

Figure S1. Morphological findings in S1 and S2 muscle biopsies.
Panels A and B: electron microscopy of S1 muscle showing mitochondria with cristae disarray and osmiophilic inclusions. Scale bar: 0.3 µm.
Panel C: normal SDH histochemical reaction in S1 muscle. Scale bar: 100 µm.
Panel D: normal haematoxylin and Eosin (H & E) reaction in S1 muscle. Scale bar: 100 µm.
Panel E: normal SDH histochemical reaction in S2 muscle. Scale bar: 100 µm.
Panel F: normal haematoxylin and Eosin (H & E) reaction in S2 muscle. Scale bar: 100 µm.

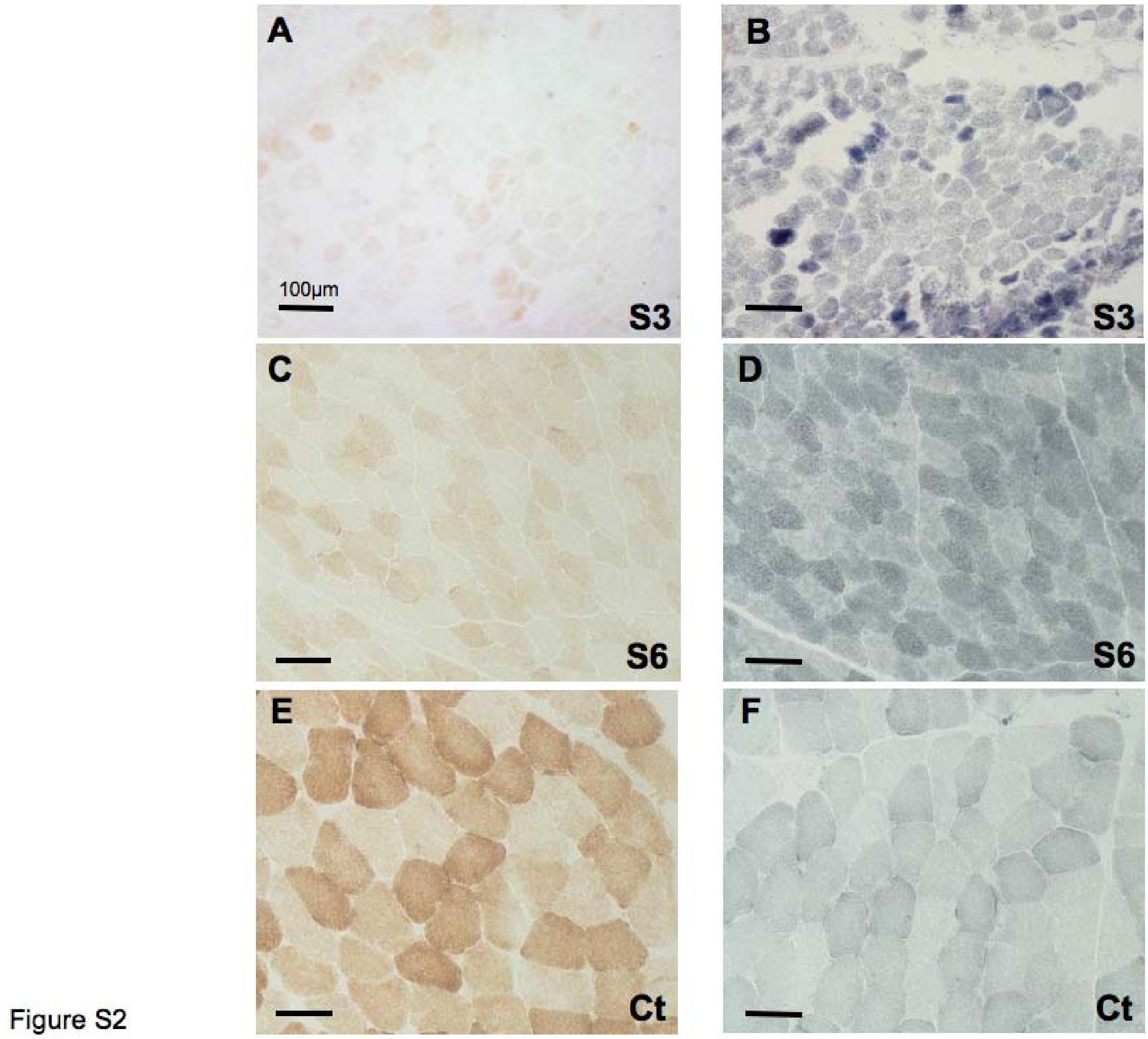

Figure S2. Additional morphological findings in muscle biopsies.
Panels A and B: COX and SDH/COX histochemical reactions in S3. Note the profound generalized decrease of the COX reaction. The SDH reaction is essentially normal. Scale bar: 100 µm.
Panels C and D: COX and SDH histochemical reactions in S6. Note the profound generalized decrease of the COX reaction. The SDH reaction is essentially normal. Scale bar: 100 µm.
Panels E and F: COX and SDH histochemical reactions in a control muscle (Ct). Scale bar: 100 µm.

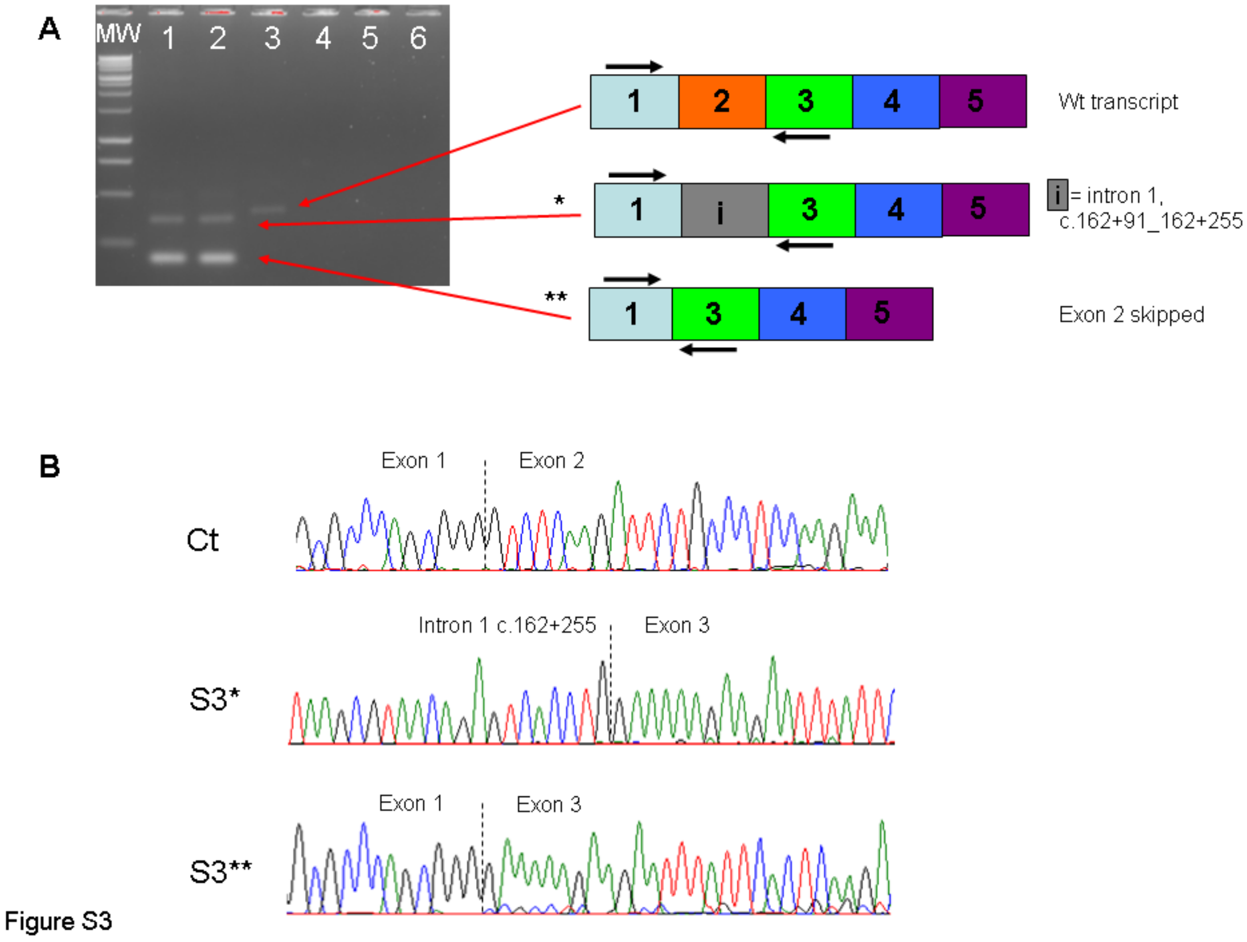

Figure S3. Characterization of the c.163-1G>A *APOPT1* mutation in S3.
Panel A: Eth-Br stained bands corresponds to PCR amplifications and schematic representation of the splicing errors identified in of the *APOPT1* cDNA from S3. In the ethidium-bromide stained agarose gel, lanes 1 and 2 show two independent PCR amplifications of the mutant cDNA from S3 muscle. Lane 3 show the PCR amplification obtained from wt control muscle. Lanes 4, 5, 6 are negative controls (corresponding to genomic DNA, RT negative and PCR negatives, respectively). The interpretation of the band composition is given in a schematic representation of the transcripts amplified by the same primer pair flanking exon 2 (black arrows). In the mutant, two aberrant cDNA fragments are PCR amplified: a 317 bp band corresponding to the partial retention of intron 1 and the skipping of exon 2 (*); a 153 bp band corresponding to the skipping of exon 2 (**); a single 350 bp band containing exon 2 is amplified from the wt cDNA (Ct).
Panel B: the Sanger' sequence of the PCR bands confirm the scheme shown in Panel A.

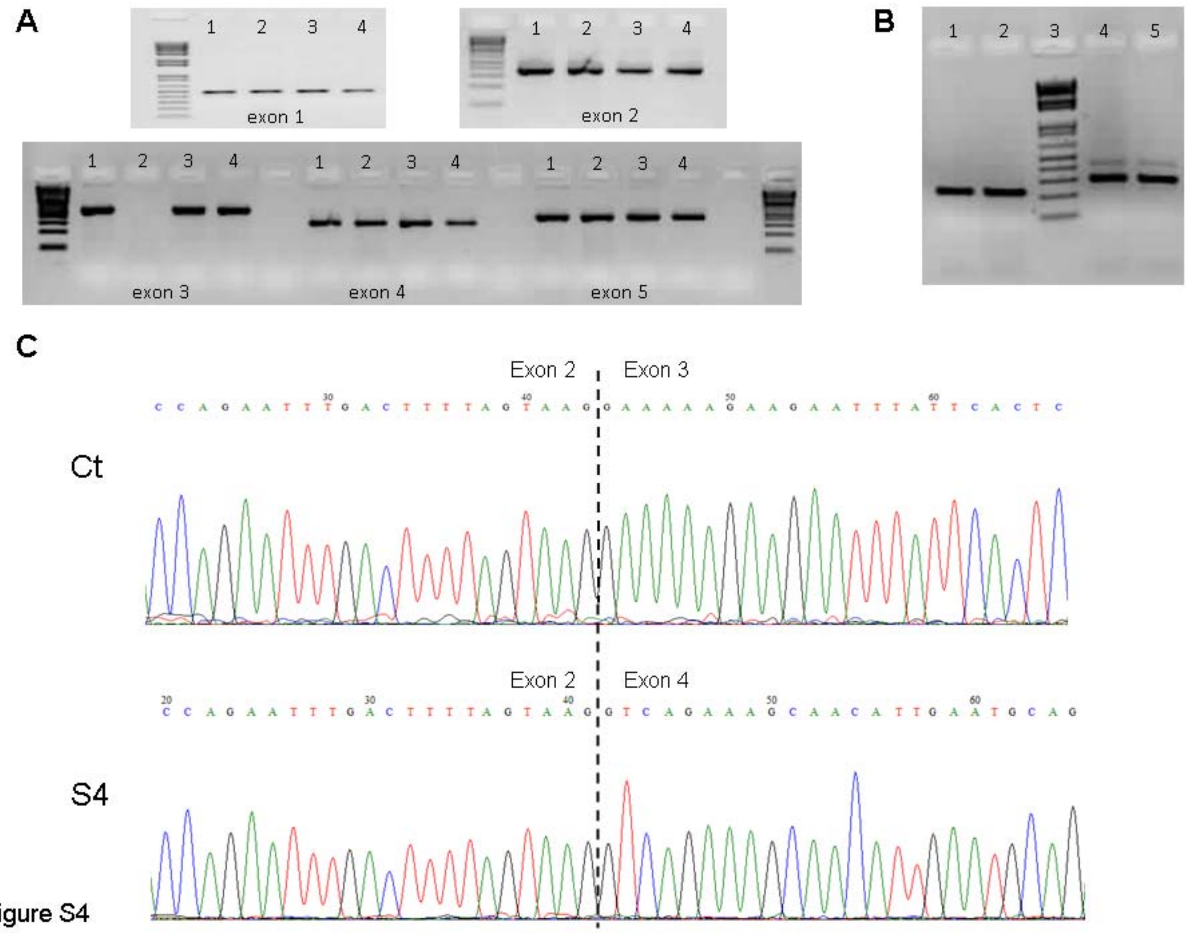

Figure S4

Figure S4. Characterization of the Exon 3 deletion of *APOPT1* in S4.
Panel A: PCR amplification of individual exons of *APOPT1* in control DNAs (lanes 1, 3), in S4 (lane 2) and S5 (lane 4). No band corresponding to exon 3 is amplified from S4 DNA. The other exons are normally amplified.
Panel B: PCR amplification of *APOPT1* cDNAs from S4 (pre-treated or not with cycloheximide; lanes 1 and 2 respectively) and control (pre-treated or not with cycloheximide; lanes 4 and 5 respectively) fibroblasts. Lane 3 is the DNA marker (1kb plus, Invitrogen). The band amplified from S4 corresponds to a cDNA fragment smaller than that amplified from the controls (see main text for details).
Panel C: Sequence analysis of the bands shown in Panel B confirmed the absence of Exon 3 in S4.

A

```
C.elegans        50  AREELNQWNSDFWAEHNQLFDRQKSDFVERK--------QQELGRLEHVS   91
D.melanogaster   84  KRIEVEAWNTDFWTKHNKRFYEEKEDFIR---------LHKESGT-SEVS  123
D.rerio          92  LRQETEDWNHEFWTNQNFTFNKEKEEYIQSQLSAKGLSERDDDGRKRTLS  141
G.gallus         77  LREETQAWNQSFWARQNTAFQREKEEFIYSRLKARGLEARDETGQKVTLS  126
M.musculus       87  LRQETQEWNQQFWAKQNLSFNKEKEEFIYSRLQAKGAGLRTESGQRATLD  136
R.norvegicus     86  LRQETQEWNQQFWAKQNLSFNKEKEEFIYSRLQAKGSGPRTESGQRATLD  135
B.taurus         85  LRQETQEWNQQFWADQNLTFHKEKEEFVRSRLKAKGLDLRTASGQKATLN  134
C.lupus          54  LRQETQEWNQQFWANQNLTFRKEKEEFIHSRLKAKGLELRSGSGQKATLD  103
M.mulatta        99  LRQETQEWNQQFWANQNLTFSKEKEEFIHLRLKTKGLGLRTESGQKATLN  148
H.sapiens        99  LRQETQEWNQQFWANQNLTFSKEKEEFIHSRLKTKGLGLRTESGQKATLN  148
```

B

Tools	Prediction	Score	Notes
Polyphen2	Probably damaging	1.000	Maximal deleteriousness=1
SIFT	Affect protein function	0	probabilities < .05 are predicted to be deleterious
Mutation Taster	Disease causing	0.9999	Probability of the prediction
Pmut	Pathological	0.8360	Maximal deleteriousness=1

C

S5
c.353T>C

S5 father

S5 mother

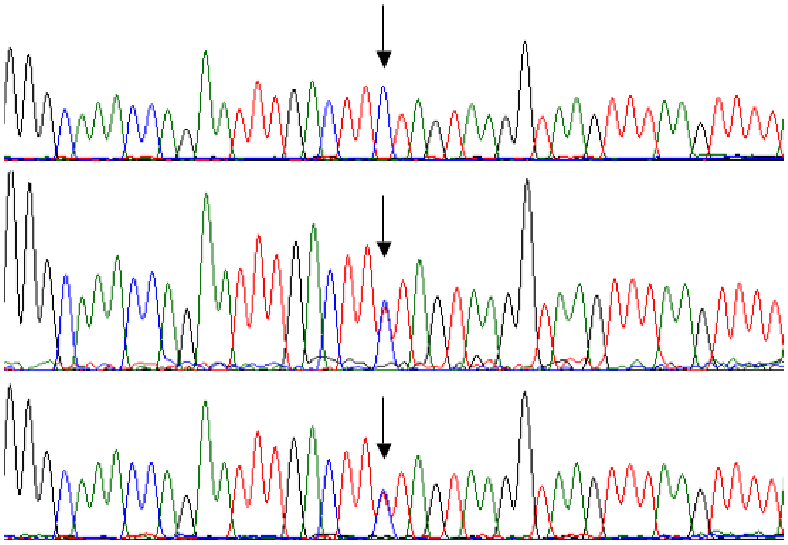

Figure S5

Figure S5. Characterization of the APOPT1 Phe118Ser mutation in S5.
Panel A: phylogenetic conservation of the Phe118 residue (highlighted in yellow) throughout species. Single letter aminoacid code.
Panel B: in silico analysis of pathogenicity using different prediction tools.
Panel C: Sequence analysis of the mutation containing *APOPT1* gene in S5 and parents. The c.353T>C change is indicated by an arrow.

A

Tools	Subcellular localization	Score	Predicted MTS
Mitoprot	mitochondrion	92%	39aa from Met1; 26aa from Met14
TargetP	mitochondrion	86%	39aa from Met1; 26aa from Met14
Softberry	mitochondrion	67%	/
Bacello	mitochondrion	/	/

B

```
D.melanogaster 54%  1  ------------MNKCFR----CQPRISLFQFSLPRCYAAVQPGCPPPQ  33
D.rerio        75%  1  ------MNVETARYLIFRSLRPCYTNTNFSLRSPCRGHRESTSQAPKHA  43
G.gallus       36%  1  -------MAAVGAARALRAGGCRYSR-FSSSFSSGGGERAGP-------  34
M.musculus     99%  1  -------------MAALRPGSRALRRLLCRSFSGGGGVRLARERPTDHR  36
R.norvegicus   99%  1  -------------MAALRPGSKALRRLLCRSFS-GGGVRLARERATERR  35
B.taurus       99%  1  --------------MAPRRPAKRLVTALCRAFS-GRGCQLAPKRGAERR  34
M.mulatta      98%  1  -MLPCAAGARGRGAMVALRAGKKSFLRALSRSFV-CRGCQLAPERGAERR 48
H.sapiens      92%  1  -MLPCAAGARGRGAMVVLRAGKKTFLPPLCRAFA-CRGCQLAPERGAERR 48
                        M1           M14
```

C

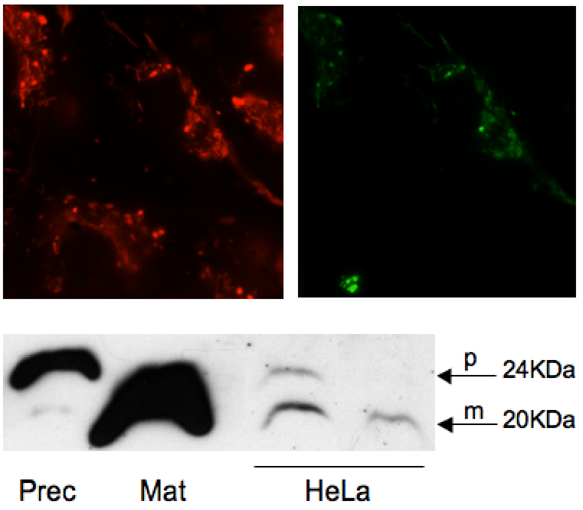

Figure S6

Figure S6. Analysis of the APOPT1 N-terminal sequence
Panel A: in silico prediction of subcellular localization using different prediction tools.
Panel B: phylogenetic analysis of the APOPT1 N-terminus. Methionine 1 (M1) is present only in humans and apes, whereas methionine 14 (M14) is invariant throughout the species analysed.
Panel C: the green fluorescent pattern obtained by transient expression of an APOPT1-HA protein starting from M1 (left), coincides with pattern obtained with Mitotracker red (center), producing a yellow pattern by overlay.
Panel D: APOPT1-HA (starting from M14) transiently expressed in HeLa cells is visualized as two faint bands, p and m (arrows) of 24 and 20 kDa, corresponding to the in vitro translated APOPT1 precursor (Prec) and mature (Mat) protein species. The mature protein is produced by cleaving off an approximately 4 kDa N-terminal MTS from the precursor species.

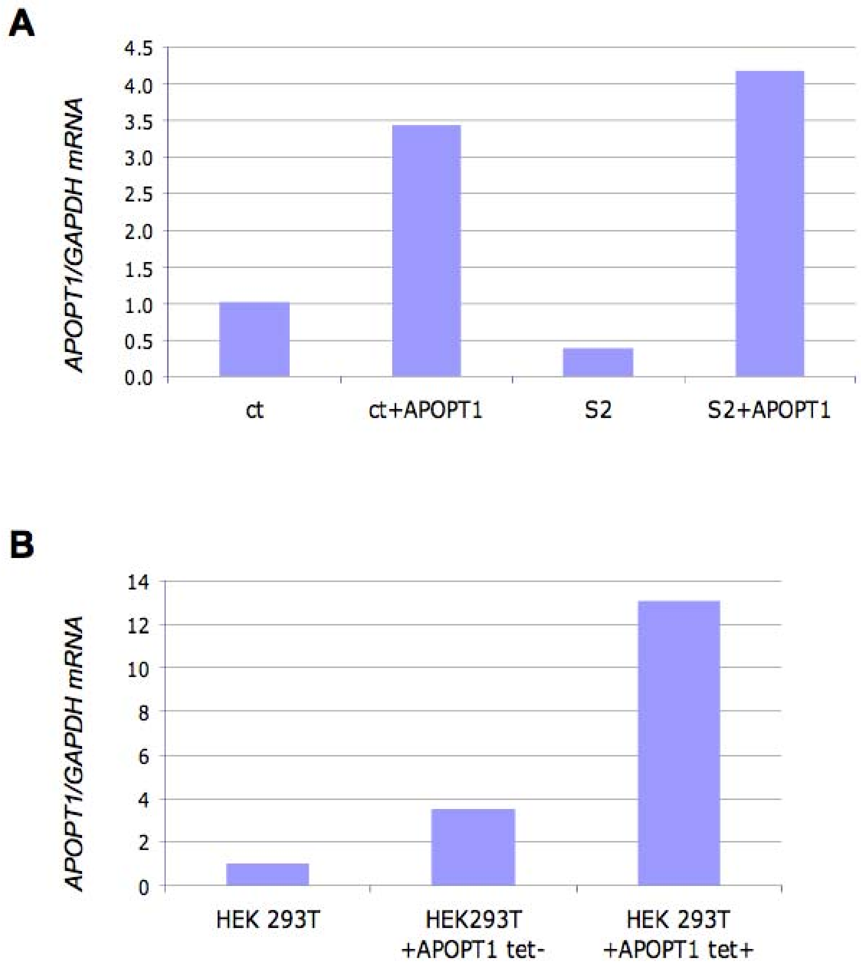

Figure S7

Figure S7. Expression of *APOPT1* in transduced/transfected cell lines

Panel A. Quantitative PCR analysis of *APOPT1* transcript in immortalized fibroblasts from subject 2 (S2) and a control individual (Ct), in basal conditions or stably transduced with *APOPT1-HA* (+APOPT1). The values of *APOPT1* transcript were normalized to *GAPDH*.

Panel B. Quantitative PCR analysis of *APOPT1* transcript in untreated HEK293T cells or in cells transfected with a Tet on-off inducible vector, expressing the *APOPT1-HA* transcript (+APOPT1), grown either without (tet-) or with 1μg/ml doxycycline (tet+).

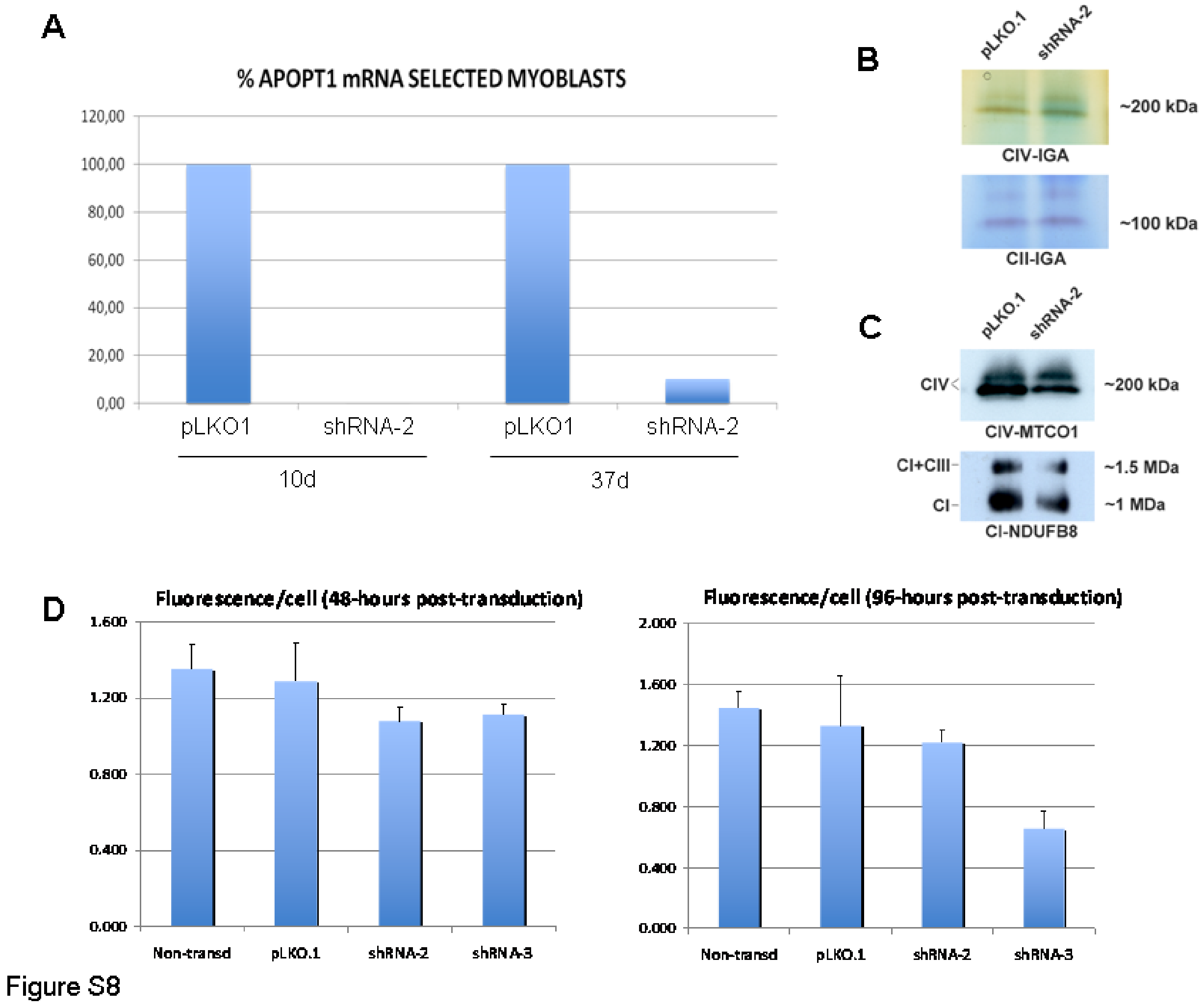

Figure S8. shRNA knockdown of *APOPT1* mRNA
Panel A. Quantitative PCR analysis of *APOPT1* transcript in myoblasts transduced with the "empty" vector pLKO.1, and with *APOPT1* specific shRNA-2. 10d: RNA extracted 10 days post-infection; 37d: 37 days post-infection.
Panel B. In-gel Activity (IGA) for complex IV (CIV) and II (CII) in myoblasts transduced with the "empty" vector pLKO.1 and with *APOPT1* specific shRNA-2. Samples were taken at 37 days post-infection and solubilized with 1% dodecylmaltoside.
Panel C. Western-blot analysis of one-dimension BNGE in myoblasts transduced with the "empty" vector pLKO.1 and with *APOPT1* specific shRNA-2. Samples were taken at 37 days post-infection and solubilized with 1% dodecylmaltoside. We used an antibody against MTCO1 for complex IV (CIV) and an antibody against NDUFB8 to detect complex I (I).
Panel D. ROS detection by dichlorofluorescein (DCHF, Invitrogen) fluorescence on naïve, non-transduced immortalized fibroblasts, fibroblasts transduced with the "empty" vector pLKO.1, and with *APOPT1* specific shRNA-2 and shRNA-3, under exposure to 100uM H_2O_2. Bars represent standard deviations.

Table S1 MRI findings

Subject	S1	S2	S3	S4	S5	S6
Age early MRI (years)	no early MRI available	no early MRI available	4	5	5	3
Cerebral WM abn. - frontal<br>- parietal<br>- occipital<br>- temporal			small lesions in frontal and temporal WM, severe abn. parieto-occipital WM	lesions in R frontal periventricular WM, severe abn. parieto-occipital WM	tiny lesions in frontal and temporal WM, severe abn. parieto-occipital WM	small lesions in frontal and temporal WM, severe abn. parieto-occipital WM
Predominant location WM abn.<br>- lobe<br>- zone			parieto-occipital, periventricular and deep WM	parieto-occipital, periventricular and deep WM	parieto-occipital, periventricular and deep WM	parieto-occipital, periventricular and deep WM
Cysts in abnormal WM			countless	countless	countless	countless
Corpus callosum abn.- genu<br>- body<br>- splenium			entire corpus callosum affected, posterior part most severely	posterior half of corpus callosum severely affected, anterior half spared	tiny lesion in genu; splenium and posterior part body severely affected	one lesion in genu, splenium severely affected, middle segment spared
Internal capsule abn. - anterior<br>- posterior			only posterior limb	only posterior limb	only posterior limb	only posterior limb
Aspect WM abnormalities			inhomogeneous, confluent and multifocal	inhomogeneous, confluent and multifocal	inhomogeneous, confluent and multifocal	inhomogeneous, confluent
Abn. cerebral cortex, basal nuclei, thalami			-	-	-	-
Enlargement lateral ventricles			mild	-	-	-
Enlargement subarachnoid spaces			-	-	-	-
Abn. cerebellar WM and peduncles			-	-	-	-
Abn. cerebellar cortex, dentate nucleus			-	-	-	-
Cerebellar atrophy			-	-	-	-
Abn. midbrain			-	-	parieto-occipito-pontine tracts	parieto-occipito-pontine tracts
Abn. pons			-	-	-	-
Abn. Medulla			-	pyramids	-	-
Contrast enhancement			in multiple foci of abnormal WM	in multiple foci of abnormal WM	in multifocal areas of abnormal WM	no evident enhancement
Restricted diffusion			not investigated	in multifocal areas of abnormal WM	in multifocal areas of abnormal WM	in multifocal areas of abnormal WM
MRS lactate			highly elevated in abnormal WM	highly elevated in abnormal WM	not investigated	highly elevated in abnormal WM

Subject	S1	S2	S3	S4	S5	S6
Age at follow-up MRI (years)	**21**	**15**	**10**	9½	no follow-up MRI	no follow-up MRI
Cerebral WM abn. - frontal<br>- parietal<br>- occipital<br>- temporal	all cerebral WM highly atrophic and abn. signal throughout	only signal abn. in parieto-occipital WM	abn. signal throughout, but most severe in parieto-occipital WM	lesion in R frontal periventricular WM, severe abn. parieto-occipital WM		
Predominant location WM abn.<br>- lobe<br>- zone	no predominance	parieto-occipital, periventricular and deep WM	parieto-occipital, periventricular and deep WM	parieto-occipital, periventricular and deep WM		
WM cysts	multiple small cysts	multiple small cysts	countless, but partially collapsed	countless, but partially collapsed		
Corpus callosum abn.- genu<br>- body<br>- splenium	highly atrophic and abn. in signal throughout	only lesion in splenium	affected throughout and highly atrophic	posterior half of corpus callosum affected, anterior half spared		
Internal capsule abn. - anterior<br>- posterior	only posterior limb	-	only posterior limb	only posterior limb		
Aspect WM abnormalities	confluent	confluent	inhomogeneous, confluent	inhomogeneous, confluent, multifocal		
Abn. cerebral cortex, basal nuclei, thalami	-	-	-	-		
Enlargement lateral ventricles	severe	-	mild	mild		
Enlargement subarachnoid spaces	mild	-	slight	-		
Abn. cerebellar WM and peduncles	subcortical cerebellar WM	-	-	-		
Abn. cerebellar cortex, dentate nucleus	-	-	-	-		
Cerebellar atrophy	+	-	-	-		
Abn. midbrain	pyramidal tracts	-	pyramidal and parieto-occipito-pontine tracts	pyramidal and parieto-occipito-pontine tracts		
Abn. pons	-	-	-	-		
Abn. Medulla	-	-	-	pyramids		
Contrast enhancement	not investigated	not investigated	not investigated	not investigated		
Restricted diffusion	not investigated	not investigated	not investigated	-		
MRS lactate	elevated	not elevated	elevated	not elevated		
Change over time			stabilization and atrophy of abn. areas, collapse of cysts, no new abn.	stabilization and atrophy of abn. areas, collapse of cysts, no new abn., normalization of lactate		

WM, white matter; abn., abnormal/abnormality/abnormalities; -, absent
