# Supplementary material for: Mutations in APOPT1, Encoding a Mitochondrial Protein, Cause Cavitating Leukoencephalopathy with Cytochrome c Oxidase Deficiency
Source: Am J Hum Genet. 2014 Sep 4;95(3):315–25. doi: 10.1016/j.ajhg.2014.08.003 (PMC4157140; doi:10.1016/j.ajhg.2014.08.003)
Supplement: Document S2. Article plus Supplemental Data [file mmc2.pdf]

Mutations in *APOPT1*, Encoding a Mitochondrial Protein, Cause Cavitating Leukoencephalopathy with Cytochrome *c* Oxidase Deficiency

Laura Melchionda,[1] Tobias B. Haack,[2,3] Steven Hardy,[4] Truus E.M. Abbink,[5] Erika Fernandez-Vizarra,[6] Eleonora Lamantea,[1] Silvia Marchet,[1] Lucia Morandi,[7] Maurizio Moggio,[8] Rosalba Carrozzo,[9] Alessandra Torraco,[9] Daria Diodato,[1,9] Tim M. Strom,[2,3] Thomas Meitinger,[2,3] Pinar Tekturk,[10] Zuhal Yapici,[10] Fathiya Al-Murshedi,[11] René Stevens,[12] Richard J. Rodenburg,[13] Costanza Lamperti,[1] Anna Ardissone,[14] Isabella Moroni,[14] Graziella Uziel,[14] Holger Prokisch,[2,3] Robert W. Taylor,[4] Enrico Bertini,[9] Marjo S. van der Knaap,[5] Daniele Ghezzi,[1,15,*] and Massimo Zeviani[1,6,15,*]

Cytochrome *c* oxidase (COX) deficiency is a frequent biochemical abnormality in mitochondrial disorders, but a large fraction of cases remains genetically undetermined. Whole-exome sequencing led to the identification of *APOPT1* mutations in two Italian sisters and in a third Turkish individual presenting severe COX deficiency. All three subjects presented a distinctive brain MRI pattern characterized by cavitating leukodystrophy, predominantly in the posterior region of the cerebral hemispheres. We then found *APOPT1* mutations in three additional unrelated children, selected on the basis of these particular MRI features. All identified mutations predicted the synthesis of severely damaged protein variants. The clinical features of the six subjects varied widely from acute neurometabolic decompensation in late infancy to subtle neurological signs, which appeared in adolescence; all presented a chronic, long-surviving clinical course. We showed that APOPT1 is targeted to and localized within mitochondria by an N-terminal mitochondrial targeting sequence that is eventually cleaved off from the mature protein. We then showed that APOPT1 is virtually absent in fibroblasts cultured in standard conditions, but its levels increase by inhibiting the proteasome or after oxidative challenge. Mutant fibroblasts showed reduced amount of COX holocomplex and higher levels of reactive oxygen species, which both shifted toward control values by expressing a recombinant, wild-type *APOPT1* cDNA. The shRNA-mediated knockdown of *APOPT1* in myoblasts and fibroblasts caused dramatic decrease in cell viability. *APOPT1* mutations are responsible for infantile or childhood-onset mitochondrial disease, hallmarked by the combination of profound COX deficiency with a distinctive neuroimaging presentation.

Cytochrome *c* oxidase (COX, complex IV [cIV], E.C. 1.9.3.1) is the terminal component of the mitochondrial respiratory chain (MRC), operating the electron transfer from reduced cytochrome *c* to molecular oxygen. The redox reaction is coupled with proton translocation across the inner mitochondrial membrane, thus contributing to the formation of the mitochondrial membrane electrochemical potential ($\Delta\Psi$). $\Delta\Psi$ is eventually utilized by the F_1F_0-ATP synthase (complex V) to produce ATP, the universal energy currency of the cell. Human COX is composed of several subunits:[1,2] the three largest are encoded by mitochondrial DNA (mtDNA) genes and form the catalytic core of the enzyme. The remaining 11 nuclear-encoded subunits, some of which have tissue-specific isoforms,[3] are deemed to play an ill-defined regulatory role.

COX deficiency (MIM 220110) is one of the most common biochemical abnormalities found in mitochondrial disorders, but about half of all cases remain genetically undefined.[4] Mutations in mtDNA or nuclear DNA genes encoding COX subunits are exceptionally rare, suggesting that direct damage of the structural components of cIV is likely to cause embryonic lethality in most cases. Conversely, early-onset COX deficiency is often due to mutations in assembly factors of the enzyme,[5] *SURF1* (MIM 185620) being the most commonly affected gene.[6,7] *SURF1* mutant individuals typically present with Leigh syndrome (LS), an early-onset, rapidly progressive encephalopathy characterized by bilateral focal necrotizing lesions in the basal ganglia and brainstem nuclei. In addition, a number of mutations in genes involved in mtDNA

[1]Unit of Molecular Neurogenetics, Foundation IRCCS Institute of Neurology Besta, 20126 Milan, Italy; [2]Institute of Human Genetics, Technische Universität München, Munich 81675, Germany; [3]Institute of Human Genetics, Helmholtz Zentrum München, Neuherberg 85764, Germany; [4]Wellcome Trust Centre for Mitochondrial Research, Institute of Neuroscience, The Medical School, Newcastle University, Newcastle upon Tyne NE1 7RU, UK; [5]Departments of Child Neurology and Functional Genomics, Neuroscience Campus Amsterdam, VU University and VU University Medical Center, Amsterdam 1081 HV, the Netherlands; [6]MRC Mitochondrial Biology Unit, Cambridge CB2 0XY, UK; [7]Neuromuscular Diseases and Neuroimmunology Unit, Foundation IRCCS Institute of Neurology Besta, 20133 Milan, Italy; [8]Neuromuscular Unit, Department of Neurology, Centro Dino Ferrari, Fondazione IRCCS Ca' Granda Ospedale Maggiore Policlinico, University of Milan, 20122 Milan, Italy; [9]Unit of Neuromuscular Disorders, Laboratory of Molecular Medicine, Bambino Gesu' Children's Research Hospital, 00165 Rome, Italy; [10]Department of Neurology, Istanbul Faculty of Medicine, Istanbul University, 34098 Istanbul, Turkey; [11]Genetic and Developmental Medicine Clinic, Sultan Qaboos University Hospital, Muscat 123, Oman; [12]Department of Paediatrics, CHC Clinique de l'Espérance at Liège, Liège 4000, Belgium; [13]Nijmegen Center for Mitochondrial Disorders, Laboratory for Genetic, Endocrine, and Metabolic Disorders, Department of Pediatrics, Radboud University Medical Center, 9101 Nijmegen, the Netherlands; [14]Department of Child Neurology, Foundation IRCCS Institute of Neurology Besta, 20133 Milan, Italy
[15]These authors contributed equally to this work
*Correspondence: dghezzi@instituto-besta.it (D.G.), mdz21@mrc-mbu.cam.ac.uk (M.Z.)
http://dx.doi.org/10.1016/j.ajhg.2014.08.003. ©2014 The Authors
This is an open access article under the CC BY license (http://creativecommons.org/licenses/by/3.0/).

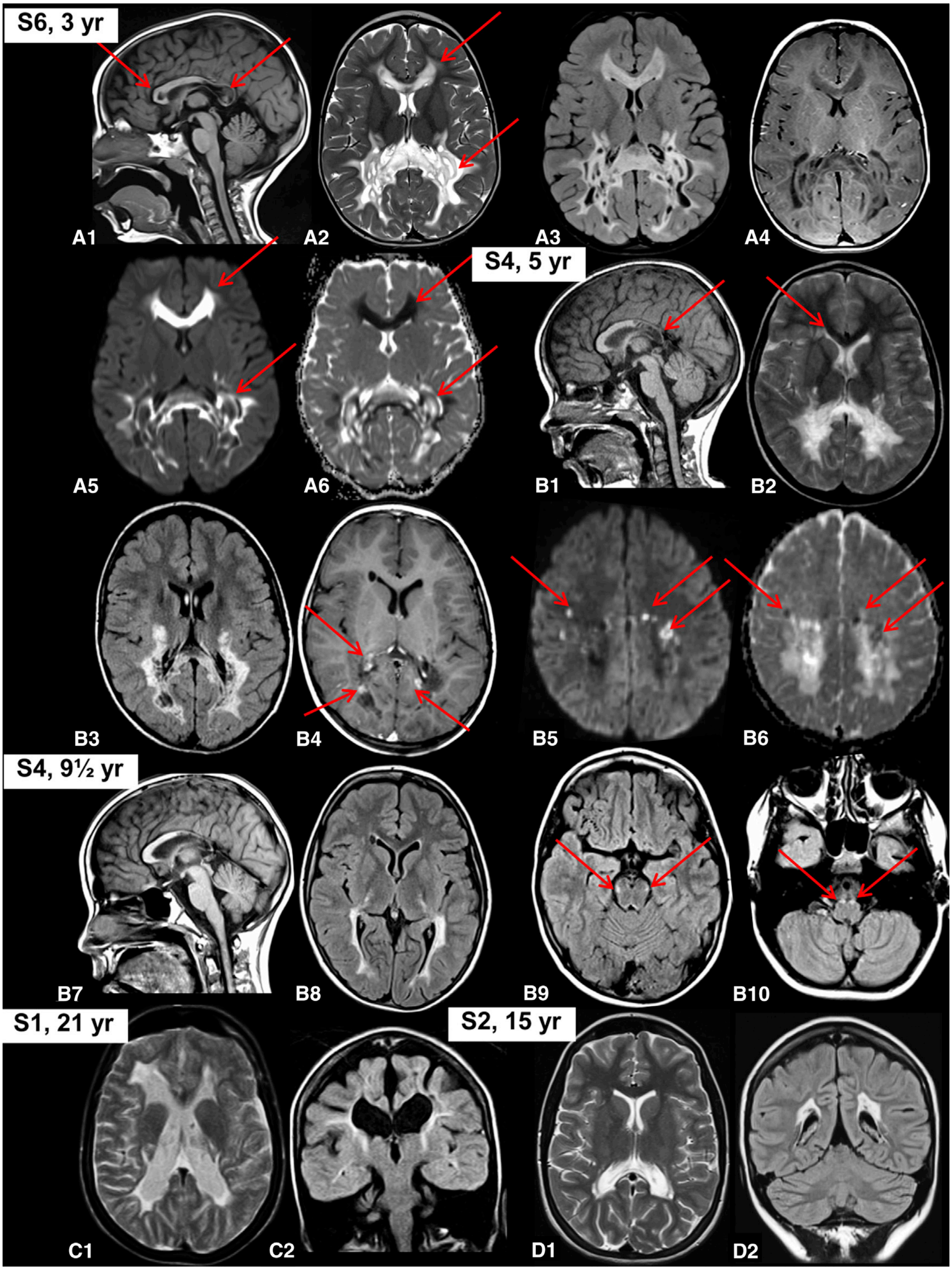

Figure 1. MRI Findings

(A) MRI abnormalities observed in individual S6 in the acute stage at the age of 3 years. The sagittal image shows signal abnormalities in the posterior part of the corpus callosum and a single lesion at the genu (red arrows in A1). Axial T2-weighted (A2, red arrows), FLAIR (A3), and T1-weighted (A4) images show signal abnormalities predominantly involving the posterior part of the cerebral white matter and corpus callosum with numerous small and larger, well-delineated cysts. The diffusion-weighted images show that the noncavitated abnormalities have a high signal, suggesting diffusion restriction (red arrows in A5), as confirmed by the low signal on the apparent diffusion coefficient (ADC) maps (red arrows in A6).

(B) MRI abnormalities observed in individual S4 in the subacute stage at the age of 5 years. The sagittal image shows the involvement of the posterior part of the corpus callosum (red arrow in B1). Axial T2-weighted (B2), FLAIR (B3), and T1-weighted (B4) images show signal abnormalities predominantly involving the posterior part of the cerebral white matter and corpus callosum with numerous small, well-delineated cysts. Additional minor abnormalities are seen next to the anterior horn of the lateral ventricle on the right (red arrows in B2

(legend continued on next page)

expression or translation are consistently associated with isolated or predominant COX deficiency,[8–11] including some mutations in mitochondrial tRNA-encoding genes or in nuclear-encoded mtDNA translation proteins (e.g., *LRPPRC* [MIM 607544] or several mitochondrial aminoacyl tRNA synthetases). As part of a long-standing project aimed at identifying novel genes responsible for COX deficiency, we present here the identification of deleterious mutations in *APOPT1* (Apoptogenic-1, previously *APOP-1* or *C14ORF153*), encoding a mitochondrial protein. This gene was identified by whole-exome sequencing (WES) analysis in three individuals from independent cohorts of subjects with isolated COX deficiency and subsequently in three additional unrelated children on the basis of a distinctive brain MRI pattern present in all.

Informed consent for participation in this study was obtained from the parents of all investigated subjects, in agreement with the Declaration of Helsinki and approved by the Ethical Committees of the Centers participating in this study, where biological samples were obtained.

A total of six individuals from five families were found to harbor mutations in *APOPT1* (see below). The clinical features varied widely from acute neurometabolic decompensation in late infancy to subtle neurological signs presenting in adolescence. Encephalopathic episodes were characterized by acute loss of developmental milestones including ability to walk or sit, loss of speech, episodes with somnolence and seizure, and pyramidal signs rapidly evolving into spastic tetraparesis. In all cases, the clinical course subsequently tended to stabilize and in several subjects marked recovery of neurological milestones was observed over time. Brain MRI was characterized by a cavitating leukodystrophy, predominantly involving the posterior cerebral white matter and the corpus callosum in the acute stage, after which the abnormalities partially improved and then stabilized (Figure 1). A summary of the clinical features is presented in Table 1 (for further details contact the corresponding authors). The MRI features are summarized in Table S1 available online and a detailed description is provided in the legend of Figure 1.

Histological examination[12] of muscle biopsies from individual S1, taken at 2.5 years, and from individual S2, taken at 7 months of age, demonstrated diffuse, profound reduction of histochemical COX reaction (Figures 2A and 2B), compared to a control muscle (Figure 2C). EM studies on the muscle biopsy from individual S1 showed the presence of enlarged mitochondria with osmiophilic inclusions and disorganization of the cristae (Figures 2D, S1A, and S1B).

Biochemical analysis of individual MRC complex activities[13] of individual S1 muscle homogenate showed that cIV activity, normalized to citrate synthase (CS), was 20% of the mean normal value in muscle and 61% in fibroblasts. A partial decrease in complex II activity (cII/CS) was also noted in muscle (44%) and fibroblasts (58%); however, spectrophotometric succinate dehydrogenase (SDH) activity was normal in both tissues and the histochemical SDH reaction in muscle was also normal (Figures S1C and S1D). The SDH reaction in the individual S2 muscle biopsy was normal as well (Figures S1E and S1F). Biochemical assay of individual S2 muscle homogenate revealed marked increase of CS activity in muscle homogenate, resulting in reduced values of all the respiratory chain activities when normalized to CS. Nevertheless, cIV/CS showed the most severe defect in muscle (3% of the controls' mean). Additionally, a partial decrease of cII/CS and cIV/CS activities was detected in fibroblasts. Histochemical and biochemical analyses of a muscle biopsy from individual S3 performed at age 10 years showed profound COX deficiency, with a residual cIV/CS activity of 5% of the controls' mean (Figures S2A and S2B, Table 2); fibroblasts were not available for further study. In muscle and fibroblasts obtained from individual S4 at 5 years, a severe decrease in cIV/CS activity (8% and 25%, respectively) was found. Individual S6 muscle biopsy taken at 2 years showed diffuse reduction of COX histochemical activity (Figures S2C and S2D), and spectrophotometric analysis of respiratory chain enzymes showed isolated cIV/CS defect (36% of the control mean). Furthermore, the histochemical reaction to COX was dramatically decreased in S6 fibroblasts (Figure 2E) compared to a control cell line (Figure 2F). A summary of the MRC activities is provided in Table 2 for all cases with the exception of individual S5 who did not undergo investigative muscle or skin biopsies.

Mutations in *SURF1* and mtDNA were excluded in individuals S1, S3, S4, and S6. Southern blot analysis showed no deletion or depletion of individual S1 muscle mtDNA, although the elevated CS activity in individual S2 muscle was accompanied by a 3-fold increase in mtDNA content[14] compared to age-matched control muscle specimens (not shown). WES was subsequently performed on DNA from individuals S1 and S2;[15] after filtering steps to exclude common SNPs (frequency > 0.2%), we searched for homozygous or compound heterozygous variants shared by the two sisters, according to a predicted recessive mode of inheritance. From the list of genes prioritized by this procedure, we then selected (1) variants known to be associated with MRC defects and (2) novel recessive variants affecting genes that encode known or predicted mitochondrial proteins.[15] As a result, a homozygous variant was identified in *APOPT1*, a gene on chr14q32.33 (Table 3,

and B4). After contrast, enhancement of multiple foci is seen (red arrows in B4). The diffusion-weighted images show multiple small foci of high signal, suggesting diffusion restriction (red arrows in B5), as confirmed by the low signal on the ADC maps (red arrows in B6). Follow-up MRI of the same subject (B7–B10) shows striking improvement (B7 and B8). Involvement of long tracts within the brain stem is now visible (red arrows in B9 and B10).
(C) Late follow-up MRI of individual S1 at age 21 shows atrophy and gliosis of what is remaining of the cerebral white matter (C1) with some small cysts in the abnormal white matter (C2).
(D) MRI of individual S2 shows only minor posterior cerebral white matter abnormalities at age 15 (D1) with tiny cysts (D2).

Table 1. Clinical Features

Affected Subject	S1	S2	S3	S4	S5	S06
Gender	female	female	male	male	male	female
Year of birth	1987	1998	1997	2000	2007	2009
Siblings (affected / unaffected / otherwise affected)	1 / 0 / 0	1 / 0 / 0	0 / 1 / 0	0 / 1 / 0	0 / 8 / 0	0 / 1 / 0
Consanguinity parents	−	−	+	+	+	−
Pregnancy, delivery, neonatal period	normal	normal	normal	normal	normal	normal
Initial motor & cognitive development	normal	normal	normal	normal	mildly delayed	normal
**Single Episode of Regression**						
Age at presentation (years)	2.5	never developed neurological signs	3	5	5	2
Signs at presentation	L-hemiparesis, somnolence, irritability, loss of ambulation	NA	delayed speech, gait difficulties	gait difficulties	dysarthria and gait difficulties	frequent falls and leg weakness
Preceding event	recurrent vomiting and poor growth	NA	NA	NA	febrile illness	febrile illness
Signs of regression	severe spastic tetraparesis, lowered consciousness	NA	spastic tetraparesis L > R	spastic tetraparesis, ataxia and sensorimotor polyneuropathy with loss of unsupported walking	spastic tetraparesis, ataxia and sensorimotor polyneuropathy with loss of unsupported walking	spastic tetraparesis and sensorimotor polyneuropathy with loss of ambulation; gastrostomy due to swallowing defect
Treatment	temporary improvement on steroids; riboflavin, coenzyme Q10, thiamine and vitamin C	coenzyme Q10, carnitine and vitamin C	none	none	none	riboflavin, coenzyme Q10
Duration of regression	2 months	NA	2 years	2–3 months	2–3 months	5 months
Further regressions	seizures at age 4, controlled with carbamazepine; no further regression	NA	a somnolence episode with generalized seizure at age 5; 3 seizures during follow-up of 11 years; no further regression	no further regression	no further regression	no further regression
**Outcome**						
Age (years)	26	14	16	13	6.5	4
Motor function	wheelchair-bound	normal	moderate spastic tetraparesis L > R; wheelchair-bound	walks, mild signs of spasticity, ataxia, and peripheral neuropathy	walks, mild signs of spasticity, ataxia, and peripheral neuropathy	walks, spastic gait
Cognitive level	decreased	normal	decreased	slightly decreased	normal	normal
Speech and language	single words, marked dysarthria	normal	dysarthria	normal	normal	normal

Figures 3A–3C). The c.235C>T (RefSeq accession number NM_032374.3) nucleotide substitution is predicted to introduce a stop codon causing the synthesis of a truncated protein (p.Arg79*; RefSeq NP_115750.2). This mutation was confirmed by Sanger sequencing in both individuals S1 and S2, and the parents were shown to be heterozygous carriers.

WES was independently performed on individual S3, identifying a nucleotide change c.163−1G>A (chr14: 104,037,959 G>A) in *APOPT1* by the same filtering

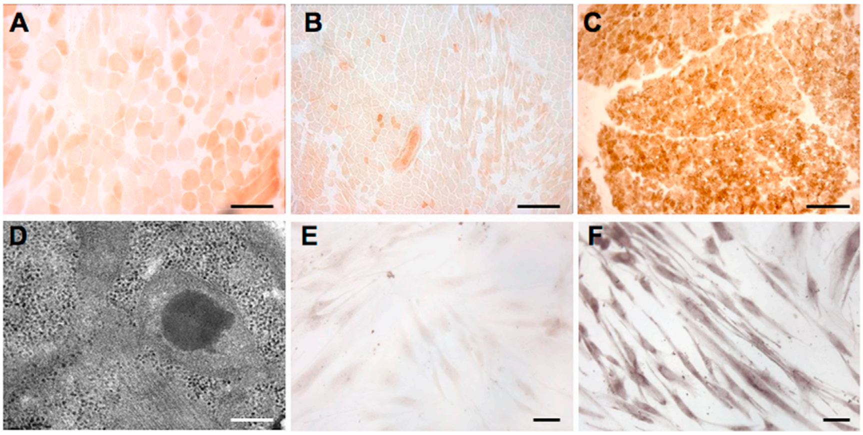

Figure 2. Morphological Findings
(A–C) The histochemical reaction to COX is diffusely decreased in muscle biopsies of individual S1 (A) and individual S2 (B), compared to a control biopsy (C). Scale bars represent 100 μm.
(D) Electron microscopy of muscle from individual S1 shows abnormal mitochondria with osmiophilic inclusions and cristae disarray. Scale bar represents 0.3 μm.
(E and F) Profound decrease of COX histochemical reaction is also visualized in fibroblasts from individual S6 (E) compared to a control cell line (F). Scale bars represent 10 μm.

strategy (Table 3, Figures 3A–3C). This variant is within the conserved consensus splice acceptor site of intron 1. Using muscle-derived individual S3 cDNA to study *APOPT1* transcripts, we showed that exon 2 is completely skipped in the majority of transcripts, predicting the maintenance of the open reading frame for the synthesis of a 140-amino-acid-long species lacking approximately one-third of the wild-type protein (p.Val55_Lys120del). Low-level transcripts appeared to show partial retention of intron 1 (c.162+91_162+255) (Figure S3). No trace of normal *APOPT1* mRNA was detected by this analysis.

We then sequenced *APOPT1* in five subjects characterized by cavitating leukoencephalopathy with posterior predominance, and found mutations in three individuals (S4, S5, and S6). Individuals S4 and S6 presented with severe COX deficiency whereas individual S5 was not investigated biochemically. Additional subjects with isolated cIV deficiency with or without unspecific leukoencephalopathic changes ($n = 10$) were also screened, but no further mutations were identified.

PCR amplification of exon 3 of *APOPT1* was unsuccessful using genomic DNA from individual S4 (Figure S4A), suggesting a homozygous deletion of the corresponding genomic region, and no mutation was identified in other exons. Since we successfully generated PCR products of exons 2 and 4, we assume that the deletion does not extend beyond 15,328 bp, corresponding to the distance between oligonucleotide primers 2R and 4F. Accordingly, analysis of the cDNA retrotranscribed from the mutant transcript showed the absence of the mRNA portion encoded by exon 3 (Figures S4B and S4C). The deletion of exon 3 causes a change in the reading frame of *APOPT1* and is predicted to result in the introduction of a premature stop codon (p.Glu121Valfs*6). In individual S5, we found a homozygous c.353T>C mutation transition, predicting a p.Phe118Ser substitution. Phe118 is highly conserved, with mutation to a serine residue being predicted as extremely deleterious by several bioinformatics tools (Figure S5). In individual S6, we identified two heterozygous mutations: the same c.235C>T change present in individuals S1 and S2 and a three-nucleotide deletion (c.370_372delGAA) causing the elimination of a highly conserved amino acid residue (p.Glu124del). Parents were shown to be heterozygous carriers of one mutation, and a healthy sibling was heterozygous for the nonsense mutation. Details of the *APOPT1* mutations and corresponding changes in the protein are summarized in Figure 3A and Table 3.

APOPT1 is predicted to be a mitochondrial protein possessing an N-terminal mitochondrial targeting signal (MTS) (Figure S6A). Two putative ATG start codons are present in the open reading frame NM_032374.3, encoding methionines at positions 1 and 14; however, the predicted mature forms of APOPT1 precursors starting from Met1 (APOPT1-M1) or Met14 (APOPT1-M2) are the same, because cleavage is predicted to occur between amino acids 39 and 40 (Figure S6B). GFP-tagged recombinant murine APOPT1 was previously demonstrated to have mitochondrial localization when transiently expressed in cultured cells.[16] Using suitable recombinant constructs inserted into lentiviral vectors (pLenti6.3/V5-TOPO vector system, Invitrogen), we showed that both the human GFP-tagged APOPT1-M1 and APOPT1-M2 proteins colocalize with a mitochondrial marker (Mitotracker red) when transiently transduced in fibroblast cells (Figures 3D and S6C). However, we considered the 193-amino-acid sequence starting from M14 as the most likely human APOPT1 protein, for two reasons. First, although the APOPT1 sequence is conserved in animals, M1 is absent in all species except primates (Figure S6B). Second, the human *APOPT1* transcript (RefSeq NM_032374.3) has only one nucleotide in the 5′ UTR upstream of the first AUG, and it is known that ribosomes do not recognize start codons that are less than 12–20 nucleotides downstream of the cap structure in the 5′ UTR. Therefore, we used APOPT1-M2 (named APOPT1 hereafter) for all further experiments.

We tested diverse commercial antibodies against human APOPT1 (Abcam, Santa Cruz) but none showed clear immunoreactivity by either immunoblot or immunofluorescence. We therefore created a lentiviral vector encoding a recombinant human APOPT1 protein tagged with the 9-amino-acid-long HA epitope at the C terminus (APOPT1-HA). Using an anti-HA monoclonal antibody,

Table 2. **Mitochondrial Respiratory Chain Activities**

	Subject	cI/CS[a]	cII/CS[a]	cIII/CS[a]	cIV/CS[a]	CS[b]
Muscle biopsy	S1	142	44*	91	20*	90
	S2	47*	11*	32*	3*	374*
	S3	140	69	93	5*	119
	S4	58	100	42*	8*	29*
	S6	127	81	132	36*	100
Skin biopsy	S1	128	58	105	61	101
	S2	56	54*	82	50*	147
	S4	58	98	104	25*	150*

The analyses were performed in different laboratories, and the reference values are diverse (usually ranging between 60% and 150% of the mean control value). The values out of the control range (specific for each enzymatic activity and for each laboratory) are indicated with an asterisk (*).
[a]Percent of mean control value of MRC complexes/citrate synthase (CS) activities.
[b]Percent of mean control value.

we performed immunoblot analysis on lysates of transiently transduced HeLa cells.[17] We detected two faint immunoreactive bands with the same electrophoretic mobility of the in vitro synthesized[17] putative APOPT1-HA precursor (193+9 amino acids, predicted MW 24 kDa) and mature (167+9 amino acids, predicted MW 20 kDa) species (Figure S6D). These results confirm that human APOPT1 has an N-terminal mitochondrial targeting sequence (MTS) of ~4 kDa, which is cleaved from the mature protein species following import into the inner mitochondrial compartment.

In order to study the effect of the protein in a cellular system, we attempted to examine APOPT1-HA in HeLa and fibroblast cell lines, by transducing a recombinant lentiviral expression construct that requires puromycin as a selectable marker.[17] Although we detected high levels of recombinant *APOPT1-HA* transcript after selection (Figure S7A), hardly any protein was immunovisualized by immunoblot or immunofluorescence in either transduced cell line. To test whether this result was due to selective APOPT1-HA-induced cell death, we used a Tet on-off inducible vector, expressing the *APOPT1-HA* transcript under exposure to increasing concentrations of doxycycline. However, we were unable to detect the protein in doxycycline-treated cells expressing high levels of the *APOPT1-HA* transcript (Figure S7B) and failed to observe increased cell death in induced compared to control cells. Taken together, these results indicate that the *APOPT1-HA* cDNA is expressed transcriptionally, but the corresponding protein product is rapidly degraded by a surveillance system active in standard culturing conditions. To further explore this hypothesis, immortalized fibroblasts from either individual S2 or a control subject, stably transduced with the *APOPT1-HA* lentiviral vector, were treated with MG-132 (5 μM for 24 hr), a proteasome inhibitor.[18] HA-immunoreactive bands corresponding to the precursor and mature APOPT1-HA species were clearly present in both MG-132-treated cell lines, in contrast with the absence of HA-immunoreactive band in the same cell lines under naive, untreated conditions (Figures 4A and 4B). These results strongly suggest that APOPT1 precursor protein is degraded by the proteasome system in standard culture conditions. Next, we tested whether the levels of the APOPT1 protein responded to oxidative[19] or apoptogenic[20] challenges. We exposed the same transduced cell lines to increasing concentrations of H_2O_2 (100 μM–1 mM) or to a standard concentration of staurosporine (1 μM), an inducer of apoptosis. Under conditions of oxidative stress (H_2O_2 treatment), APOPT1-HA protein increased to immunodetectable levels, with a maximum at 24 hr (Figure 4C); no protein was detected following treatment with staurosporine (data not shown). In contrast to the effect of MG-132, exposure to H_2O_2 determined the predominant accumulation of the mature, intramitochondrial, presumably active APOPT1-HA species (Figure 4C).

To test the role of APOPT1-HA stabilization under oxidative stress, we measured the production of reactive oxygen species (ROS) using a dichlorofluorescein-based assay. While in basal conditions ROS levels were comparable between immortalized mutant S2 fibroblasts and control fibroblasts, after H_2O_2 incubation (100 μM or 1 mM for 3 hr) ROS levels in mutant S2 were higher than in control fibroblasts (Figure 4D). However, in S2 fibroblasts transduced with *APOPT1-HA*-expressing lentiviral vector, the amount of ROS was decreased with either treatment, being comparable to that found in control cells treated with the higher H_2O_2 concentration, suggesting a role for APOPT1 in mitochondrial response to ROS (Figure 4D).

Conversely, we obtained no clear evidence of a proapoptotic role for APOPT1 in available tissues (muscle, fibroblasts): a TUNEL assay was negative on muscle from individuals S1 and S2, no apoptotic bodies were observed by EM in individual S1 muscle, and no difference in apoptotic cells was found after staurosporine treatment in mutant versus control fibroblasts (not shown). However, we cannot exclude a selective apoptotic activation in other tissues/organs, for instance in brain white matter.

We then investigated the amount and integrity of the COX holocomplex by Blue-Native Gel Electrophoresis (BNGE) immunoblot analysis[17] of dodecylmaltoside-treated S1 and S2 fibroblasts. We found that the amount of both COX holocomplex and $cIII_2$+cIV supercomplex was clearly reduced in both mutant cells, more markedly in S2 (Figure 5A). The intensities of the bands corresponding to other individual MRC complexes, including cII, were comparable to controls. The cIV reduction was confirmed in immortalized fibroblasts from individual S2. In spite of the very low levels of recombinant APOPT1 in transduced individual S2 immortalized fibroblasts, we found a small but consistent increase in the amounts of cIV and supercomplex cIII+cIV in these cells compared to naive individual S2 cells (Figures 5B and 5C), suggesting a role for APOPT1 in cIV assembly and/or stability.

Table 3. *APOPT1* Mutations

Subject	Country of Origin	Mutations[a] DNA	Protein	State	Father/Mother	SNP Frequency[b]
S1[c]	Italy	c.235C>T	p.Arg79*	homo	F&M het[c]	Ø
S2[c]	Italy	c.235C>T	p.Arg79*	homo	F&M het	Ø
S3	Turkey	c.163−1G>A	Ex2 skipping; p.Val55_Lys120del	homo	NA	Ø
S4	Morocco	Ex3 deletion	Ex3 deletion; p.Glu121Valfs*6	homo	NA	NA
S5	Oman	c.353T>C	p.Phe118Ser	homo	F&M het	Ø
S6	Italy	c.235C>T	p.Arg79*	het	M het	Ø
		c.370_372del	p.Glu124del	het	F het	Ø

Abbreviations are as follows: F, father; M, mother; homo, homozygous; het, heterozygous; NA, not available; Ø, not reported variant.
[a]Nomenclature according to HGVS; reference cDNA sequence: RefSeq NM_032374.3.
[b]Frequency in dbSNP and EVS (Exome Variant Server) databases.
[c]S1 and S2 are sisters.

RNAi experiments were performed by lentiviral transduction of different shRNA sequences targeting the *APOPT1* mRNA (MISSION shRNA Library, Sigma). Cells transduced with the "empty" pLKO.1 vector were used as a control. Two shRNAs (shRNA-2 and shRNA-3) produced marked knockdown of *APOPT1* expression in different cell lines, having <10% of the control mRNA levels (Figures 5D and S8A). Primary myoblasts and immortalized control fibroblasts expressing shRNA-2 and shRNA-3 showed a dramatic decrease in cell viability around 5 days after the lentiviral infection (Figure 5E), while the cells transduced with the control vector were totally viable in selective medium. No shRNA-3 myoblasts remained after puromycin selection; surprisingly, however, the cells transduced with shRNA-2 that did survive failed to show reduction in COX activity or in the amount of the holocomplex (Figures S8B and S8C), and ROS levels were not modified in shRNA fibroblasts compared to controls (Figure S8D), either before (48 hr) or during (96 hr) the decline of cell growth.

Our work has relevant implications in the clinical definition of mitochondrial disorders and in the discovery of new mitochondrial homeostatic pathways. In six subjects belonging to five unrelated families, we identified five mutant alleles in *APOPT1*, encoding a mitochondrial protein of unknown function. All mutations are predicted to result in the synthesis of severely damaged protein species, due to premature stop, microdeletion, macrodeletion, aberrant splicing, or nonconservative substitution of an evolutionary invariant amino acid. In the five subjects for whom the histoenzymatic and biochemical characterization of the mitochondrial respiratory chain was possible, we documented a profound defect of COX activity, associated, in available cultured fibroblasts, with marked reduction of cIV holocomplex and cIII+cIV supercomplex species. In two siblings, a concomitant, partial reduction of cII/CS activity was also detected, which was not present in available samples from the other subjects, suggesting a secondary effect. *APOPT1* mutations were consistently associated with a peculiar brain MRI pattern characterized by early and rapid onset of cavitating white matter abnormalities, predominantly in the posterior areas of the cerebral hemispheres and corpus callosum. This pattern was specific enough to suggest the direct screening of *APOPT1* in a series of five subjects with cavitating leukodystrophy, three of whom proved to harbor recessive mutations in this gene. Interestingly, the two subjects without *APOPT1* mutations did not show COX deficiency in fibroblast or muscle samples. Taken together, these results indicate that mutations in *APOPT1* are responsible for a mitochondrial disorder characterized by marked COX deficiency and a well-characterized form of cavitating leukodystrophy. MRI hallmarks in the acute stage are white matter abnormalities containing numerous small, well-delineated cysts, predominantly in the posterior areas of the cerebral hemispheres, also involving the connecting corpus callosum. In severe cases, the white matter abnormalities extend into the frontal and temporal lobes as well as the anterior part of the corpus callosum. In the acute stage, multifocal areas on restricted diffusion and contrast enhancement are present, and within the abnormal white matter levels of lactate are high, as revealed by proton magnetic resonance spectroscopy. Posterior fossa structures are typically spared. On follow-up, atrophy of the affected areas, collapse of cysts, and disappearance of diffusion restriction and contrast enhancement occur, with a concomitant decrease in lactate levels.

In contrast to other neurological conditions defined by profound cIV impairment, such as rapidly progressive SURF1-deficient LS,[4] the clinical course of APOPT1-associated encephalopathy appears to be highly variable in severity. Although some individuals developed severe neurological signs at an early age, with motor impairment evolving into spastic quadriparesis, epilepsy, and severe cognitive impairment (e.g., individual S1), others showed an intermediate phenotype with severe motor impairment

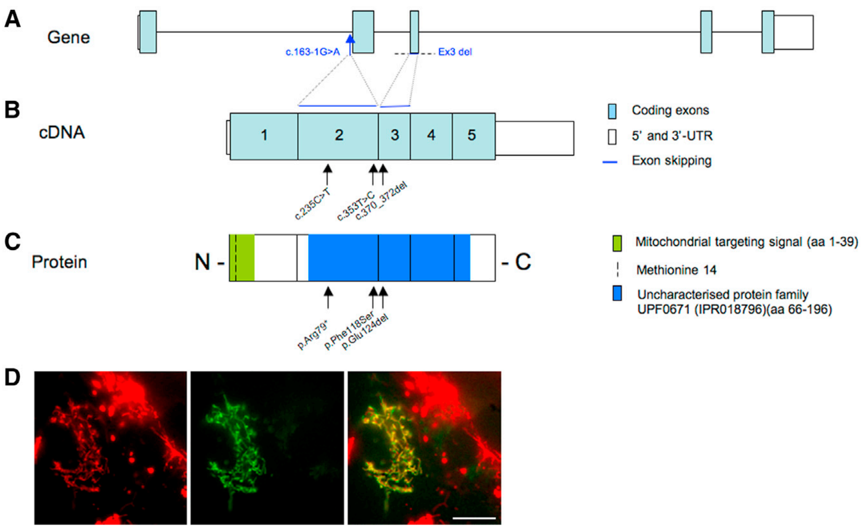

Figure 3. *APOPT1* Mutations and APOPT1 Localization
(A–C) Mutations found in this study are positioned (arrows) against schematic representations of *APOPT1* (A), cDNA (B), and protein (C).
(D) The green fluorescence pattern of an APOPT1-GFP fusion protein starting from the methionine 14, transiently expressed in control fibroblasts (center), coincide with that obtained with mitotracker red (left), to give a yellow overlay pattern (right). Scale bar represents 10 μm.

but mild or absent cognitive involvement, and in one case the neurological examination was virtually normal at 14 years of age (individual S2), despite the presence of some posterior white matter abnormalities. Strikingly, in all affected individuals who underwent electromyography and nerve conduction velocity studies, evidence of a peripheral neuropathy was also found, even in absence of clinical signs. Remarkably, severely affected individual S1 and subclinically affected individual S2 are siblings, sharing the same homozygous mutation, which predicts the synthesis of a truncated protein, again supporting the wide clinical variability of this condition. Irrespective of the severity of the onset and initial evolution, in all cases both the MRI lesions and the clinical progression stabilize, determining a chronic, long-surviving clinical course, in contrast with the rapidly downhill, often fatal outcome of other early-onset COX-defective encephalopathies. All our *APOPT1* mutant subjects are alive, some in their teens, with the oldest one being in her third decade. In some cases stabilization and improvement (individuals S1 and S6) coincided with the starting of vitamin and/or CoQ10-based treatment, and in individual S2 the administration of a vitamin cocktail since the first months of life may have helped to prevent the development of clinical symptoms. Nevertheless the efficacy of these therapies remains unproven, since other subjects (individuals S3, S4, and S5) showed stabilization with no specific treatment (Table 1).

Although white matter involvement can be an associated feature of LS, "pure" (predominant or exclusive) leukodystrophy encompasses a substantial fraction of infantile mitochondrial encephalopathies, ranging from 10% to 50% of the cases in different cohorts.[21–23] In some cases, specific MRI leukodystrophic patterns are consistently associated with mutations in specific genes related to mitochondrial function, notably a number of mitochondrial aminoacyl tRNA synthetases including *DARS2*[24] (MIM 610956), *EARS2*[25] (MIM 612799), and more recently *AARS2*[11] (MIM 612035).

APOPT1 was identified in 2006 as a new/unreported transcript in a study on murine vascular smooth muscle cells (VSMCs) cultured from atherosclerotic plaques. The gene encodes a mitochondrial protein deemed as a proapoptotic factor because its overexpression induced PTP-dependent apoptotic cell death,[16] which could be prevented by activating the Akt pathway.[26] No further published studies on this protein are available. Our experiments confirmed that APOPT1 is targeted to and localized within mitochondria. Absence of a predicted transmembrane domain and presence of a cleavable N-terminal MTS, which serves for active translocation across the inner mitochondrial membrane, suggest that APOPT1 is specifically localized in the mitochondrial matrix.

In contrast with previous studies, we obtained no clear evidence of a proapoptotic role for this protein. We observed no difference between mutant subjects and controls in both TUNEL assay in muscle and apoptotic induction with staurosporine in fibroblasts, and no massive cell death followed transient overexpression of recombinant APOPT1-HA in either HeLa cells or fibroblasts. The extremely low levels of recombinant APOPT1-HA obtained in cells grown in standard conditions, despite high levels of recombinant transcripts detected by quantitative PCR, suggest active degradation of the protein precursor, possibly via the proteasomal system. This hypothesis was supported by the stabilization of the precursor APOPT1-HA protein in recombinant cells exposed to a proteasome inhibitor.

Oxidative challenge with H_2O_2 of *APOPT1-HA*-expressing recombinant fibroblasts led to a prolonged increase in the amount of the mature protein species, suggesting a role for APOPT1 in mitochondrial anti-ROS defense mechanisms. In order to further test this hypothesis, we showed that APOPT1-deficient fibroblasts from individual S2 produce more ROS, whereas overexpression of wild-type *APOPT1* leads to a reduction of ROS. We hypothesize that APOPT1 is induced by and plays a protective role under oxidative stress conditions, but it must be otherwise eliminated in standard conditions, at least in cultured cells. A similar mechanism has been demonstrated for other proteins acting at checkpoints in mitochondrial execution pathways, for example PINK1, which is eliminated in

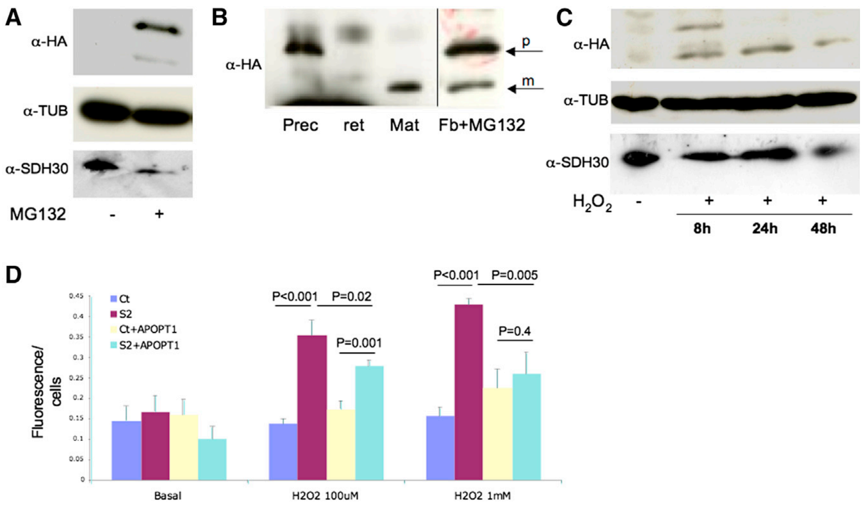

Figure 4. Functional Studies of APOPT1
(A) Immunoblot analysis of fibroblasts stably expressing APOPT1-HA. No HA-immunoreactive material, visualized using a specific anti-HA antibody (α-HA, Roche), is present in naive conditions, whereas two HA-immunoreactive bands are detected under exposure of the cells to the proteasome inhibitor MG-132. Tubulin and SDHB, immunovisualized by specific antibodies (α-TUB, Sigma-Aldrich; α-SDH30, Mitoscience), are used as loading controls.
(B) The anti-HA immunoreactive bands obtained as in (A) (arrows) have electrophoretic mobility identical to the in vitro translated cDNAs corresponding to the predicted precursor (prec) and mature (mat) APOPT1-HA protein species, synthesized using the TNT Transcription-Translation System kit (Promega). The in vitro translated products are specific, as no anti-HA immunoreactive material is visualized in the prereaction reticulocyte lysate (ret). Note that the panels are from the same filter, but different exposure times were used to better visualize the bands.
(C) Anti-HA immunoreactive bands corresponding to the precursor and mature APOPT1 species are detected in fibroblasts exposed to H_2O_2. Note that the upper band, corresponding to the precursor APOPT1-HA species, is present in the sample collected 8 hr after the exposure to H_2O_2, whereas only the mature species is detected in samples collected at 24 and 48 hr, suggesting that over time the precursor APOPT1-HA species has been translocated across the inner mitochondrial membrane and quantitatively processed into the mature species by cleavage of a 39 amino acid N-terminal MTS.
(D) ROS detection by dichlorofluorescein (DCHF, Invitrogen) fluorescence on control (Ct) and individual S2 fibroblasts in basal and oxidative stress conditions. Note that naive individual S2 fibroblasts show significantly higher levels of DCHF fluorescence under exposure to 100 μM and 1 mM H_2O_2. The levels of DCHF fluorescence are significantly lower in APOPT1-HA-expressing individual S2 fibroblasts. Bars represent standard deviations. The p values were obtained by unpaired, two-tail Student's t test.

bioenergetically proficient mitochondria, but is stabilized by the dissipation of the mitochondrial membrane potential.[27] In addition, an analogous mechanism applies to other important metabolic switch systems, such as the hypoxia program induced by HIF-1.[28]

Although COX deficiency is the biochemical hallmark of *APOPT1* mutant subjects, no mechanistic link is known between APOPT1 and cIV assembly or stability. Interestingly, a connection between apoptosis and increased ROS production has been suggested to play an important role in the pathophysiology of mitochondrial diseases associated with COX deficiency.[29,30] We showed that the expression of wild-type *APOPT1* in mutant fibroblast cells led to an increase in the amount of COX and a reduction of ROS production to normal levels. However, shRNA-mediated stable downregulation of *APOPT1* expression in human myoblasts or immortalized fibroblasts failed to either impair COX activity or increase ROS production, but was associated with markedly attenuated cell growth up to arrest of proliferation and cell degeneration. This dramatic cell growth phenotype is likely to be an acute phenomenon, consequent to the sudden suppression of *APOPT1* expression, since it was not observed in mutant fibroblasts, i.e., in a chronic condition of APOPT1 deprivation. One possibility is that shRNA-treated cells that do develop COX deficiency are rapidly eliminated, determining growth impairment, whereas the surviving cells are selected for COX proficiency.

In conclusion, *APOPT1* mutations are responsible for infantile or childhood-onset mitochondrial disease distinguished by the combination of profound, generalized deficiency in COX activity and amount, a peculiar neuroimaging presentation of cavitating leukodystrophy with posterior cerebral predominance, and a peripheral neuropathy. APOPT1 is an elusive mitochondrial protein, which appears to be actively eliminated by proteasome degradation in normal conditions of cell culturing, but be stabilized by, and participate in, a stress-induced program related to ROS production. Several questions remain open, including the high clinical variability found in mutant subjects. Future work is warranted to better characterize the conditions triggering the stabilization of APOPT1, to clarify its role in mitochondrial homeostasis, and to elucidate the mechanistic link between its impairment and a structural and functional defect of COX activity.

Supplemental Data

Supplemental Data include eight figures and one table and can be found with this article online at http://dx.doi.org/10.1016/j.ajhg.2014.08.003.

Acknowledgments

We thank Carola G.M. van Berkel and Gigliola Fagiolari for their excellent technical support. We acknowledge the "Cell lines and DNA Bank of Paediatric Movement Disorders and Neurodegenerative Diseases" and the "Bank of muscle tissue, peripheral nerve, DNA and cell culture" of the Telethon Network of Genetic Biobanks (grant GTB12001J) and the EurobiobanK Network. This work was supported by Fondazione Telethon grants GGP11011

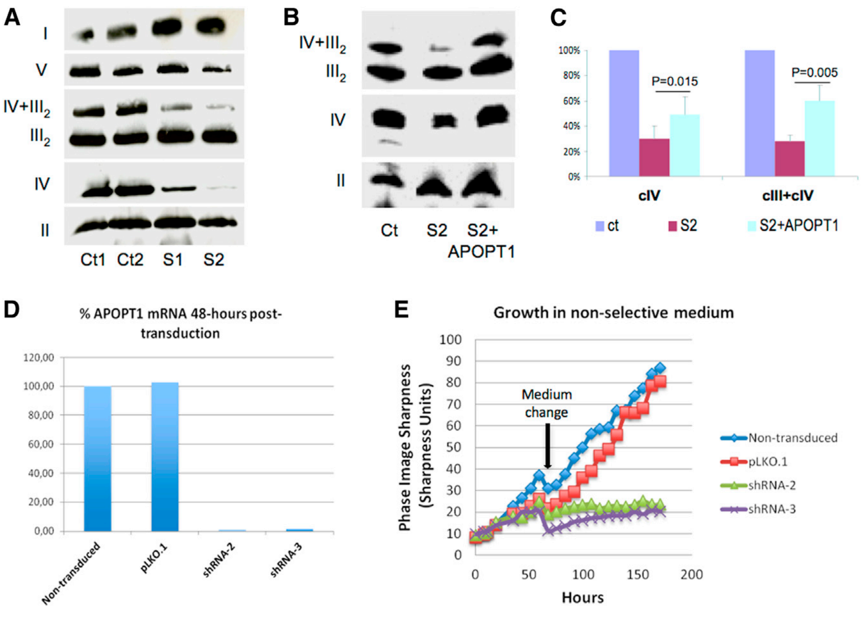

Figure 5. Complementation and RNAi Studies

(A) Immunoblot analysis of one-dimension BNGE in immortalized fibroblasts from two controls (Ct1, Ct2) and mutant subjects S1 and S2. Samples were prepared using 10% w/v dodecylmaltoside. We used an antibody against NDUFA9 to detect complex I (I), an antibody against the subunit α of ATP synthase to detect complex V (V), an antibody against SDHA for complex II (II), an antibody against core I for complex III (III), and an antibody against subunit COX4 for complex IV (IV). Note that the amounts of cIV holocomplex and cIV+cIII2 supercomplex are clearly decreased in mutant samples compared to controls.

(B) Immunoblot analysis of one-dimension BNGE in immortalized fibroblasts from a control (Ct), mutant S2, and S2 stably transduced with APOPT1-HA. Note that the amounts of cIV and cIV+$cIII_2$, which are markedly decreased in S2, are significantly increased in S2+APOPT1-HA.

(C) Quantitative densitometric analysis of the results obtained by three independent BNGE experiments. The intensities of complex IV (CIV) and supercomplex III + IV (CIII+CIV) signals were normalized to complex II; the ratio obtained in control fibroblasts was set as 100%. The p values were obtained by unpaired, two-tail Student's t test. Bars represent standard deviations.

(D) Quantitative PCR analysis of *APOPT1* transcript in naive, nontransduced immortalized fibroblasts, fibroblasts transduced with the "empty" vector pLKO.1, and with APOPT1-specific shRNA-2 and shRNA-3. The amount of *APOPT1* transcript is decreased to approximately <5% by both shRNA, compared to the amount found in naive, nontransduced cells.

(E) Growth curves in immortalized fibroblasts from a naive control cell line, and the same cell line transduced with empty vector (pLKO.1), shRNA-2, and shRNA-3. Cell lines with severe *APOPT1* knockdown (down to around 1% of the control mRNA levels 48 hr postinfection) show significantly decreased cell growth.

and GPP10005; the Italian Ministry of Health (GR2010–2316392); CARIPLO grant 2011/0526; the Pierfranco and Luisa Mariani Foundation of Italy; the Italian Association of Mitochondrial Disease Patients and Families (Mitocon); the European Research Council (ERC) Advanced Grant FP7-322424; the Impulse and Networking Fund of the Helmholtz Association in the framework of the Helmholtz Alliance for Mental Health in an Ageing Society (HA-215) and the German Federal Ministry of Education and Research (BMBF)-funded German Center for Diabetes Research (DZD e.V.) and Systems Biology of Metabotypes grant (SysMBo #0315494A), the grant RF-INN-2007-634163 of the Italian Ministry of Health, the BMBF-funded German Network for Mitochondrial Disorders (mitoNET #01GM1113C), and the E-Rare project GENOMIT (01GM1207 and FWF I 920-B13) Medical Research Council, UK. T.E.M.A. and M.S.v.d.K. were supported by the Dutch Organisation for Scientific Research (ZonMw, TOP grant 91211005). R.W.T. is funded by a Wellcome Trust Strategic Award (096919/Z/11/Z), the MRC Centre for Neuromuscular Diseases (G0601943), the Lily Foundation, and the UK NHS Highly Specialised "Rare Mitochondrial Disorders of Adults and Children" Service.

Received: May 14, 2014
Accepted: August 8, 2014
Published: August 28, 2014

Web Resources

The URLs for data presented herein are as follows:

dbSNP, http://www.ncbi.nlm.nih.gov/projects/SNP/

Human Genome Variation Society, http://www.hgvs.org/mutnomen/

Online Mendelian Inheritance in Man (OMIM), http://www.omim.org/

NHLBI Exome Sequencing Project (ESP) Exome Variant Server, http://evs.gs.washington.edu/EVS/

RefSeq, http://www.ncbi.nlm.nih.gov/RefSeq

References

1. Tsukihara, T., Aoyama, H., Yamashita, E., Tomizaki, T., Yamaguchi, H., Shinzawa-Itoh, K., Nakashima, R., Yaono, R., and Yoshikawa, S. (1996). The whole structure of the 13-subunit oxidized cytochrome c oxidase at 2.8 A. Science *272*, 1136–1144.
2. Yoshikawa, S., Shinzawa-Itoh, K., and Tsukihara, T. (1998). Crystal structure of bovine heart cytochrome c oxidase at 2.8 A resolution. J. Bioenerg. Biomembr. *30*, 7–14.
3. Arnold, S., and Kadenbach, B. (1997). Cell respiration is controlled by ATP, an allosteric inhibitor of cytochrome-c oxidase. Eur. J. Biochem. *249*, 350–354.
4. DiMauro, S., Tanji, K., and Schon, E.A. (2012). The many clinical faces of cytochrome c oxidase deficiency. Adv. Exp. Med. Biol. *748*, 341–357.
5. Ghezzi, D., and Zeviani, M. (2012). Assembly factors of human mitochondrial respiratory chain complexes: physiology and pathophysiology. Adv. Exp. Med. Biol. *748*, 65–106.
6. Tiranti, V., Hoertnagel, K., Carrozzo, R., Galimberti, C., Munaro, M., Granatiero, M., Zelante, L., Gasparini, P., Marzella, R., Rocchi, M., et al. (1998). Mutations of SURF-1 in Leigh disease associated with cytochrome c oxidase deficiency. Am. J. Hum. Genet. *63*, 1609–1621.

7. Wedatilake, Y., Brown, R.M., McFarland, R., Yaplito-Lee, J., Morris, A.A., Champion, M., Jardine, P.E., Clarke, A., Thorburn, D.R., Taylor, R.W., et al. (2013). SURF1 deficiency: a multi-centre natural history study. Orphanet J. Rare Dis. *8*, 96.
8. DiMauro, S., and Hirano, M. (2009). Pathogenesis and treatment of mitochondrial disorders. Adv. Exp. Med. Biol. *652*, 139–170.
9. Mootha, V.K., Lepage, P., Miller, K., Bunkenborg, J., Reich, M., Hjerrild, M., Delmonte, T., Villeneuve, A., Sladek, R., Xu, F., et al. (2003). Identification of a gene causing human cytochrome c oxidase deficiency by integrative genomics. Proc. Natl. Acad. Sci. USA *100*, 605–610.
10. Almalki, A., Alston, C.L., Parker, A., Simonic, I., Mehta, S.G., He, L., Reza, M., Oliveira, J.M., Lightowlers, R.N., McFarland, R., et al. (2014). Mutation of the human mitochondrial phenylalanine-tRNA synthetase causes infantile-onset epilepsy and cytochrome c oxidase deficiency. Biochim. Biophys. Acta *1842*, 56–64.
11. Dallabona, C., Diodato, D., Kevelam, S.H., Haack, T.B., Wong, L.J., Salomons, G.S., Baruffini, E., Melchionda, L., Mariotti, C., Strom, T.M., et al. (2014). Novel (ovario)leukodystrophy related to AARS2 mutations. Neurology *82*, 2063–2071.
12. Sciacco, M., and Bonilla, E. (1996). Cytochemistry and immunocytochemistry of mitochondria in tissue sections. Methods Enzymol. *264*, 509–521.
13. Bugiani, M., Invernizzi, F., Alberio, S., Briem, E., Lamantea, E., Carrara, F., Moroni, I., Farina, L., Spada, M., Donati, M.A., et al. (2004). Clinical and molecular findings in children with complex I deficiency. Biochim. Biophys. Acta *1659*, 136–147.
14. He, L., Chinnery, P.F., Durham, S.E., Blakely, E.L., Wardell, T.M., Borthwick, G.M., Taylor, R.W., and Turnbull, D.M. (2002). Detection and quantification of mitochondrial DNA deletions in individual cells by real-time PCR. Nucleic Acids Res. *30*, e68.
15. Haack, T.B., Haberberger, B., Frisch, E.M., Wieland, T., Iuso, A., Gorza, M., Strecker, V., Graf, E., Mayr, J.A., Herberg, U., et al. (2012). Molecular diagnosis in mitochondrial complex I deficiency using exome sequencing. J. Med. Genet. *49*, 277–283.
16. Yasuda, O., Fukuo, K., Sun, X., Nishitani, M., Yotsui, T., Higuchi, M., Suzuki, T., Rakugi, H., Smithies, O., Maeda, N., and Ogihara, T. (2006). Apop-1, a novel protein inducing cyclophilin D-dependent but Bax/Bak-related channel-independent apoptosis. J. Biol. Chem. *281*, 23899–23907.
17. Gai, X., Ghezzi, D., Johnson, M.A., Biagosch, C.A., Shamseldin, H.E., Haack, T.B., Reyes, A., Tsukikawa, M., Sheldon, C.A., Srinivasan, S., et al. (2013). Mutations in FBXL4, encoding a mitochondrial protein, cause early-onset mitochondrial encephalomyopathy. Am. J. Hum. Genet. *93*, 482–495.
18. Han, Y.H., Moon, H.J., You, B.R., and Park, W.H. (2009). The effect of MG132, a proteasome inhibitor on HeLa cells in relation to cell growth, reactive oxygen species and GSH. Oncol. Rep. *22*, 215–221.
19. Mocali, A., Caldini, R., Chevanne, M., and Paoletti, F. (1995). Induction, effects, and quantification of sublethal oxidative stress by hydrogen peroxide on cultured human fibroblasts. Exp. Cell Res. *216*, 388–395.
20. Ghezzi, D., Saada, A., D'Adamo, P., Fernandez-Vizarra, E., Gasparini, P., Tiranti, V., Elpeleg, O., and Zeviani, M. (2008). FASTKD2 nonsense mutation in an infantile mitochondrial encephalomyopathy associated with cytochrome c oxidase deficiency. Am. J. Hum. Genet. *83*, 415–423.
21. Moroni, I., Bugiani, M., Bizzi, A., Castelli, G., Lamantea, E., and Uziel, G. (2002). Cerebral white matter involvement in children with mitochondrial encephalopathies. Neuropediatrics *33*, 79–85.
22. Uziel, G., Ghezzi, D., and Zeviani, M. (2011). Infantile mitochondrial encephalopathy. Semin. Fetal Neonatal Med. *16*, 205–215.
23. Lebre, A.S., Rio, M., Faivre d'Arcier, L., Vernerey, D., Landrieu, P., Slama, A., Jardel, C., Laforêt, P., Rodriguez, D., Dorison, N., et al. (2011). A common pattern of brain MRI imaging in mitochondrial diseases with complex I deficiency. J. Med. Genet. *48*, 16–23.
24. Scheper, G.C., van der Klok, T., van Andel, R.J., van Berkel, C.G., Sissler, M., Smet, J., Muravina, T.I., Serkov, S.V., Uziel, G., Bugiani, M., et al. (2007). Mitochondrial aspartyl-tRNA synthetase deficiency causes leukoencephalopathy with brain stem and spinal cord involvement and lactate elevation. Nat. Genet. *39*, 534–539.
25. Steenweg, M.E., Ghezzi, D., Haack, T., Abbink, T.E., Martinelli, D., van Berkel, C.G., Bley, A., Diogo, L., Grillo, E., Te Water Naudé, J., et al. (2012). Leukoencephalopathy with thalamus and brainstem involvement and high lactate 'LTBL' caused by EARS2 mutations. Brain *135*, 1387–1394.
26. Sun, X., Yasuda, O., Takemura, Y., Kawamoto, H., Higuchi, M., Baba, Y., Katsuya, T., Fukuo, K., Ogihara, T., and Rakugi, H. (2008). Akt activation prevents Apop-1-induced death of cells. Biochem. Biophys. Res. Commun. *377*, 1097–1101.
27. Narendra, D.P., and Youle, R.J. (2011). Targeting mitochondrial dysfunction: role for PINK1 and Parkin in mitochondrial quality control. Antioxid. Redox Signal. *14*, 1929–1938.
28. Zheng, X., Ruas, J.L., Cao, R., Salomons, F.A., Cao, Y., Poellinger, L., and Pereira, T. (2006). Cell-type-specific regulation of degradation of hypoxia-inducible factor 1 alpha: role of subcellular compartmentalization. Mol. Cell. Biol. *26*, 4628–4641.
29. Di Giovanni, S., Mirabella, M., Papacci, M., Odoardi, F., Silvestri, G., and Servidei, S. (2001). Apoptosis and ROS detoxification enzymes correlate with cytochrome c oxidase deficiency in mitochondrial encephalomyopathies. Mol. Cell. Neurosci. *17*, 696–705.
30. Kadenbach, B., Arnold, S., Lee, I., and Hüttemann, M. (2004). The possible role of cytochrome c oxidase in stress-induced apoptosis and degenerative diseases. Biochim. Biophys. Acta *1655*, 400–408.

The American Journal of Human Genetics, Volume *95*

Supplemental Data

Mutations in *APOPT1*, Encoding a Mitochondrial Protein, Cause Cavitating Leukoencephalopathy with Cytochrome *c* Oxidase Deficiency

Laura Melchionda, Tobias B. Haack, Steven Hardy, Truus E.M. Abbink, Erika Fernandez-Vizarra, Eleonora Lamantea, Silvia Marchet, Lucia Morandi, Maurizio Moggio, Rosalba Carrozzo, Alessandra Torraco, Daria Diodato, Tim M. Strom, Thomas Meitinger, Pinar Tekturk, Zuhal Yapici, Fathiya Al-Murshedi, René Stevens, Richard J. Rodenburg, Costanza Lamperti, Anna Ardissone, Isabella Moroni, Graziella Uziel, Holger Prokisch, Robert W. Taylor, Enrico Bertini, Marjo S. van der Knaap, Daniele Ghezzi, and Massimo Zeviani

Supplemental Data

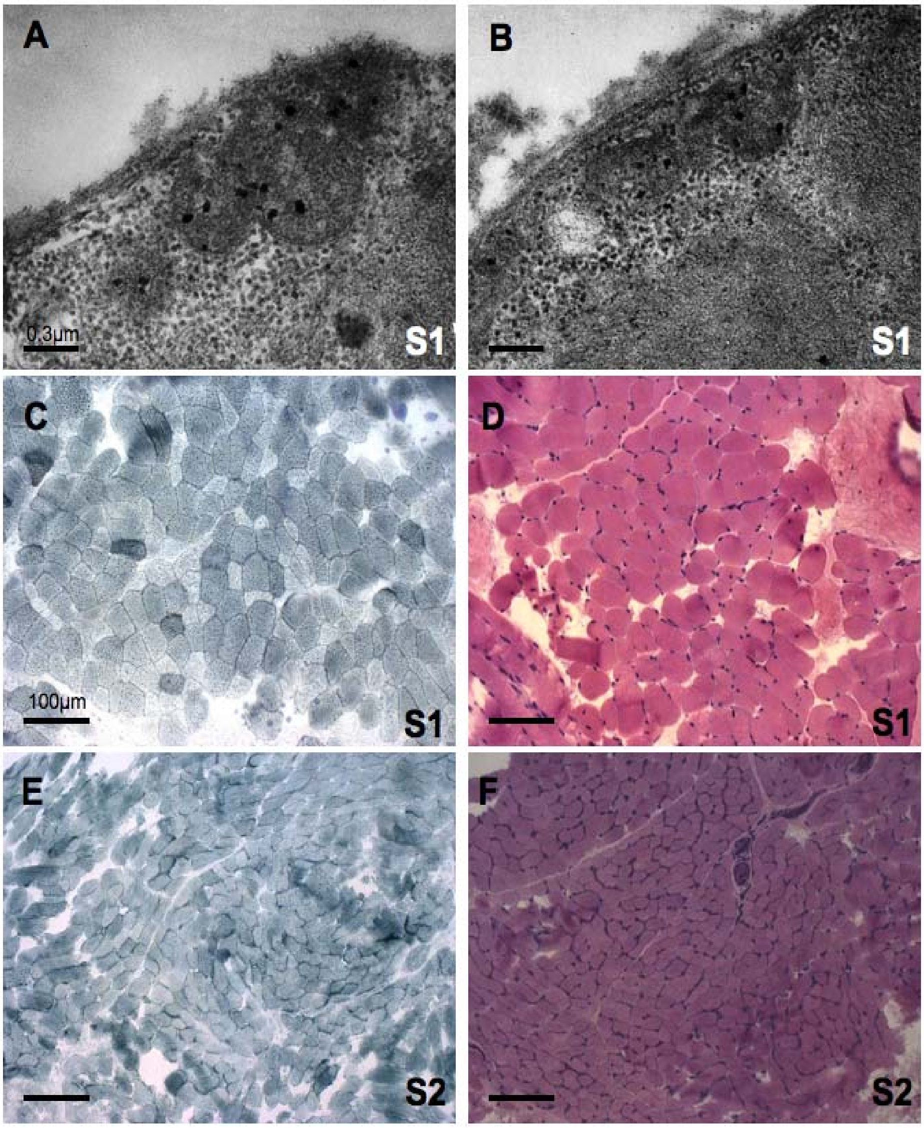

Figure S1

Figure S1. Morphological findings in S1 and S2 muscle biopsies.
Panels A and B: electron microscopy of S1 muscle showing mitochondria with cristae disarray and osmiophilic inclusions. Scale bar: 0.3 µm.
Panel C: normal SDH histochemical reaction in S1 muscle. Scale bar: 100 µm.
Panel D: normal haematoxylin and Eosin (H & E) reaction in S1 muscle. Scale bar: 100 µm.
Panel E: normal SDH histochemical reaction in S2 muscle. Scale bar: 100 µm.
Panel F: normal haematoxylin and Eosin (H & E) reaction in S2 muscle. Scale bar: 100 µm.

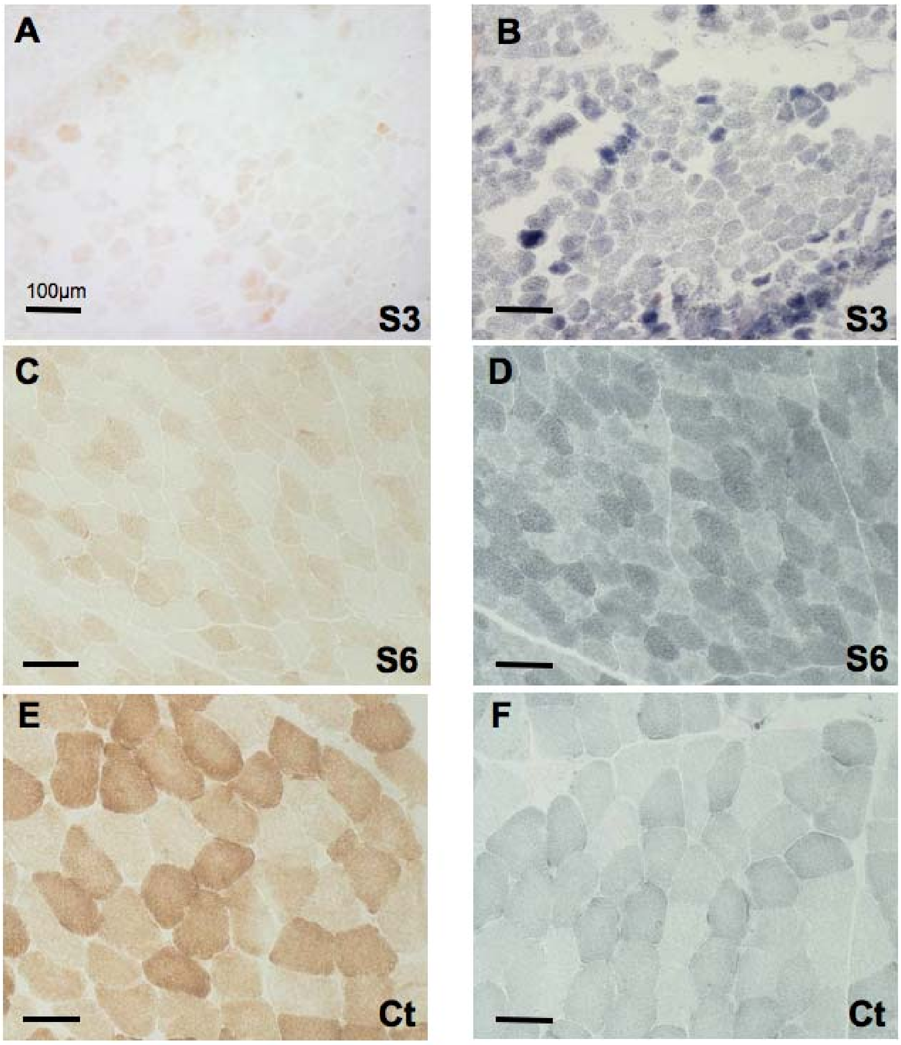

Figure S2

Figure S2. Additional morphological findings in muscle biopsies.
Panels A and B: COX and SDH/COX histochemical reactions in S3. Note the profound generalized decrease of the COX reaction. The SDH reaction is essentially normal. Scale bar: 100 µm.
Panels C and D: COX and SDH histochemical reactions in S6. Note the profound generalized decrease of the COX reaction. The SDH reaction is essentially normal. Scale bar: 100 µm.
Panels E and F: COX and SDH histochemical reactions in a control muscle (Ct). Scale bar: 100 µm.

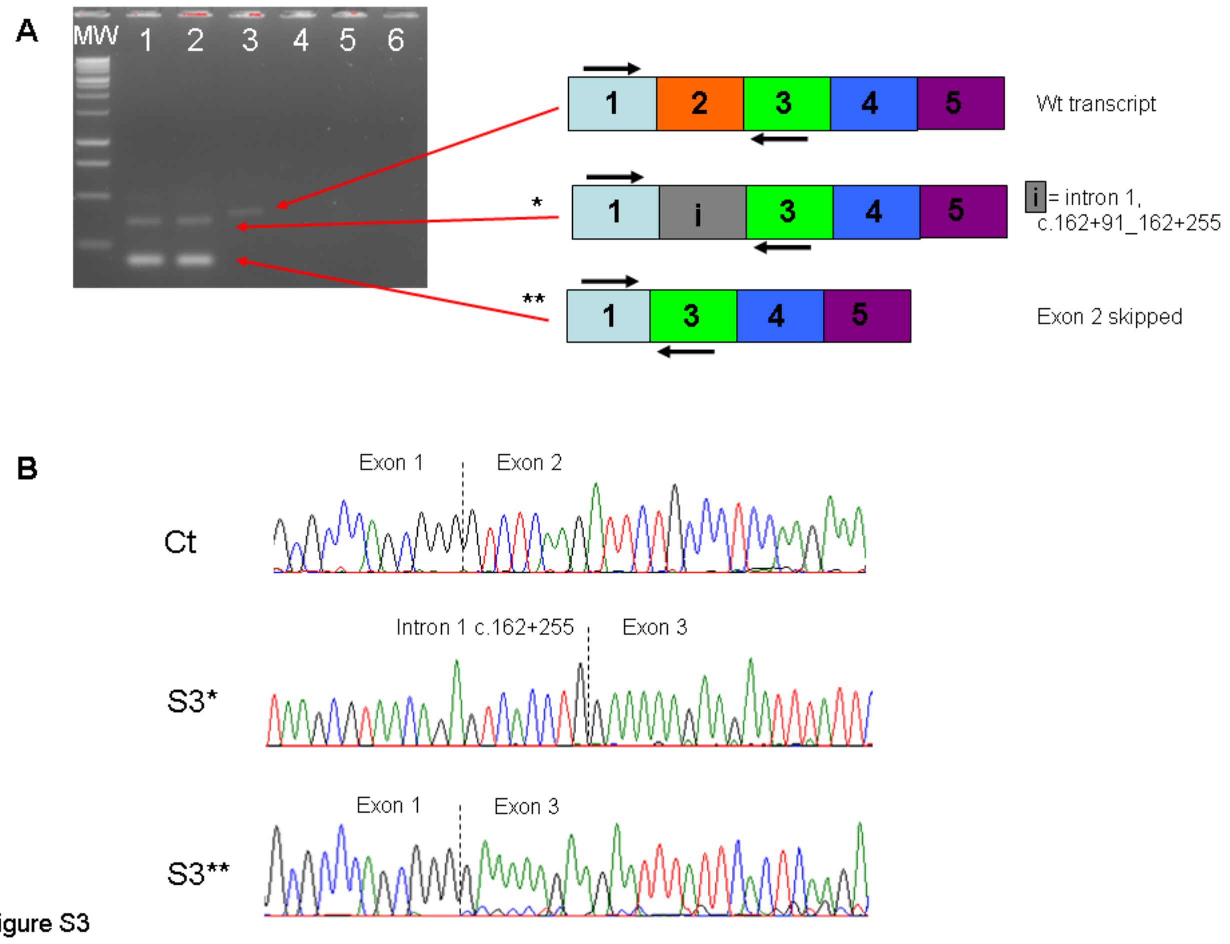

Figure S3. Characterization of the c.163-1G>A *APOPT1* mutation in S3.
Panel A: Eth-Br stained bands corresponds to PCR amplifications and schematic representation of the splicing errors identified in of the *APOPT1* cDNA from S3. In the ethidium-bromide stained agarose gel, lanes 1 and 2 show two independent PCR amplifications of the mutant cDNA from S3 muscle. Lane 3 show the PCR amplification obtained from wt control muscle. Lanes 4, 5, 6 are negative controls (corresponding to genomic DNA, RT negative and PCR negatives, respectively). The interpretation of the band composition is given in a schematic representation of the transcripts amplified by the same primer pair flanking exon 2 (black arrows). In the mutant, two aberrant cDNA fragments are PCR amplified: a 317 bp band corresponding to the partial retention of intron 1 and the skipping of exon 2 (*); a 153 bp band corresponding to the skipping of exon 2 (**); a single 350 bp band containing exon 2 is amplified from the wt cDNA (Ct).
Panel B: the Sanger' sequence of the PCR bands confirm the scheme shown in Panel A.

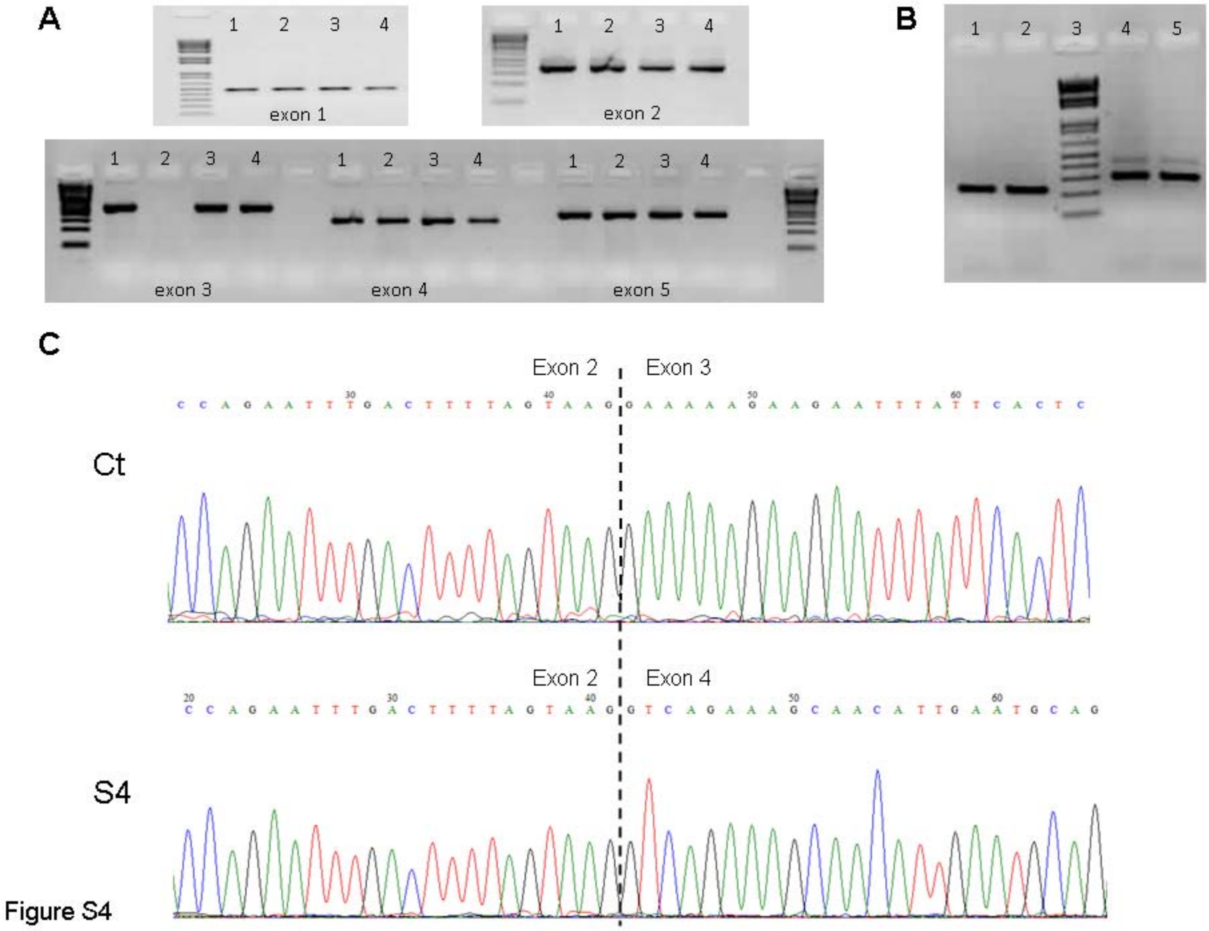

Figure S4. Characterization of the Exon 3 deletion of *APOPT1* in S4.
Panel A: PCR amplification of individual exons of *APOPT1* in control DNAs (lanes 1, 3), in S4 (lane 2) and S5 (lane 4). No band corresponding to exon 3 is amplified from S4 DNA. The other exons are normally amplified.
Panel B: PCR amplification of *APOPT1* cDNAs from S4 (pre-treated or not with cycloheximide; lanes 1 and 2 respectively) and control (pre-treated or not with cycloheximide; lanes 4 and 5 respectively) fibroblasts. Lane 3 is the DNA marker (1kb plus, Invitrogen). The band amplified from S4 corresponds to a cDNA fragment smaller than that amplified from the controls (see main text for details).
Panel C: Sequence analysis of the bands shown in Panel B confirmed the absence of Exon 3 in S4.

A

```
C.elegans         50   AREELNQWNSDFWAEHNQLFDRQKSDFVERK--------QQELGRLEHVS    91
D.melanogaster    84   KRIEVEAWNTDFWTKHNKRFYEEKEDFIR---------LHKESGT-SEVS   123
D.rerio           92   LRQETEDWNHEFWTNQNFTFNKEKEEYIQSQLSAKGLSERDDDGRKRTLS   141
G.gallus          77   LREETQAWNQSFWARQNTAFQREKEEFIYSRLKARGLEARDETGQKVTLS   126
M.musculus        87   LRQETQEWNQQFWAKQNLSFNKEKEEFIYSRLQAKGAGLRTESGQRATLD   136
R.norvegicus      86   LRQETQEWNQQFWAKQNLSFNKEKEEFIYSRLQAKGSGPRTESGQRATLD   135
B.taurus          85   LRQETQEWNQQFWADQNLTFHKEKEEFVRSRLKAKGLDLRTASGQKATLN   134
C.lupus           54   LRQETQEWNQQFWANQNLTFRKEKEEFIHSRLKAKGLELRSGSGQKATLD   103
M.mulatta         99   LRQETQEWNQQFWANQNLTFSKEKEEFIHLRLKTKGLGLRTESGQKATLN   148
H.sapiens         99   LRQETQEWNQQFWANQNLTFSKEKEEFIHSRLKTKGLGLRTESGQKATLN   148
```

B

Tools	Prediction	Score	Notes
Polyphen2	Probably damaging	1.000	Maximal deleteriousness=1
SIFT	Affect protein function	0	probabilities < .05 are predicted to be deleterious
Mutation Taster	Disease causing	0.9999	Probability of the prediction
Pmut	Pathological	0.8360	Maximal deleteriousness=1

C

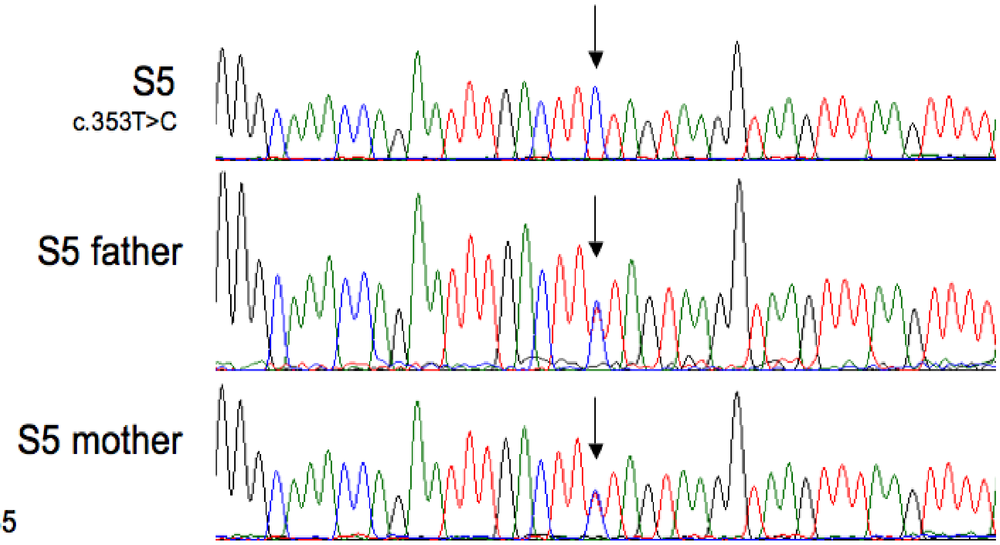

Figure S5

Figure S5. Characterization of the APOPT1 Phe118Ser mutation in S5.
Panel A: phylogenetic conservation of the Phe118 residue (highlighted in yellow) throughout species. Single letter aminoacid code.
Panel B: in silico analysis of pathogenicity using different prediction tools.
Panel C: Sequence analysis of the mutation containing *APOPT1* gene in S5 and parents. The c.353T>C change is indicated by an arrow.

A

Tools	Subcellular localization	Score	Predicted MTS
Mitoprot	mitochondrion	92%	39aa from Met1; 26aa from Met14
TargetP	mitochondrion	86%	39aa from Met1; 26aa from Met14
Softberry	mitochondrion	67%	/
Bacello	mitochondrion	/	/

B

```
D.melanogaster 54%  1   -------------MNKCFR----CQPRISLFQFSLPRCYAAVQPGCPPPQ   33
D.rerio        75%  1   -------MNVETARYLIFRSLRPCYTNTNFSLRSPCRGHRESTSQAPKHA   43
G.gallus       36%  1   --------MAAVGAARALRAGGCRYSR-FSSSFSSGGGERAGP-------   34
M.musculus     99%  1   --------------MAALRPGSRALRRLLCRSFSGGGGVRLARERPTDHR   36
R.norvegicus   99%  1   --------------MAALRPGSKALRRLLCRSFS-GGGVRLARERATERR   35
B.taurus       99%  1   ---------------MAPRRPAKRLVTALCRAFS-GRGCQLAPKRGAERR   34
M.mulatta      98%  1   -MLPCAAGARGRGAMVALRAGKKSFLRALSRSFV-CRGCQLAPERGAERR   48
H.sapiens      92%  1   -MLPCAAGARGRGAMVVLRAGKKTFLPPLCRAFA-CRGCQLAPERGAERR   48
                         M1           M14
```

C

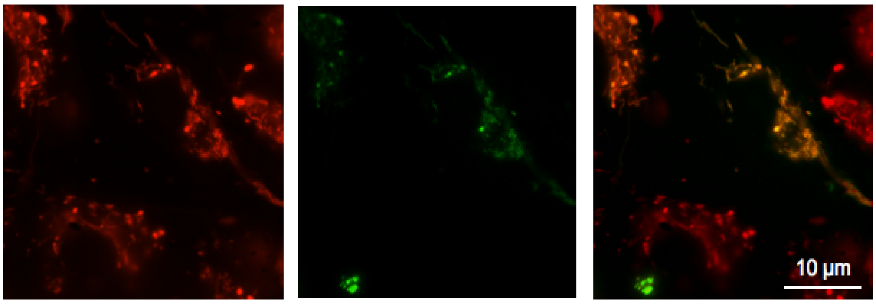

D

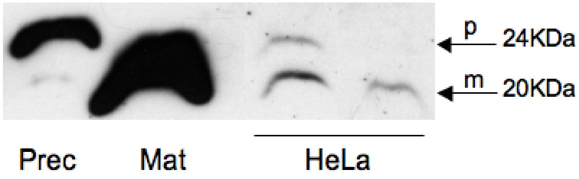

Figure S6

Figure S6. Analysis of the APOPT1 N-terminal sequence
Panel A: in silico prediction of subcellular localization using different prediction tools.
Panel B: phylogenetic analysis of the APOPT1 N-terminus. Methionine 1 (M1) is present only in humans and apes, whereas methionine 14 (M14) is invariant throughout the species analysed.
Panel C: the green fluorescent pattern obtained by transient expression of an APOPT1-HA protein starting from M1 (left), coincides with pattern obtained with Mitotracker red (center), producing a yellow pattern by overlay.
Panel D: APOPT1-HA (starting from M14) transiently expressed in HeLa cells is visualized as two faint bands, p and m (arrows) of 24 and 20 kDa, corresponding to the in vitro translated APOPT1 precursor (Prec) and mature (Mat) protein species. The mature protein is produced by cleaving off an approximately 4 kDa N-terminal MTS from the precursor species.

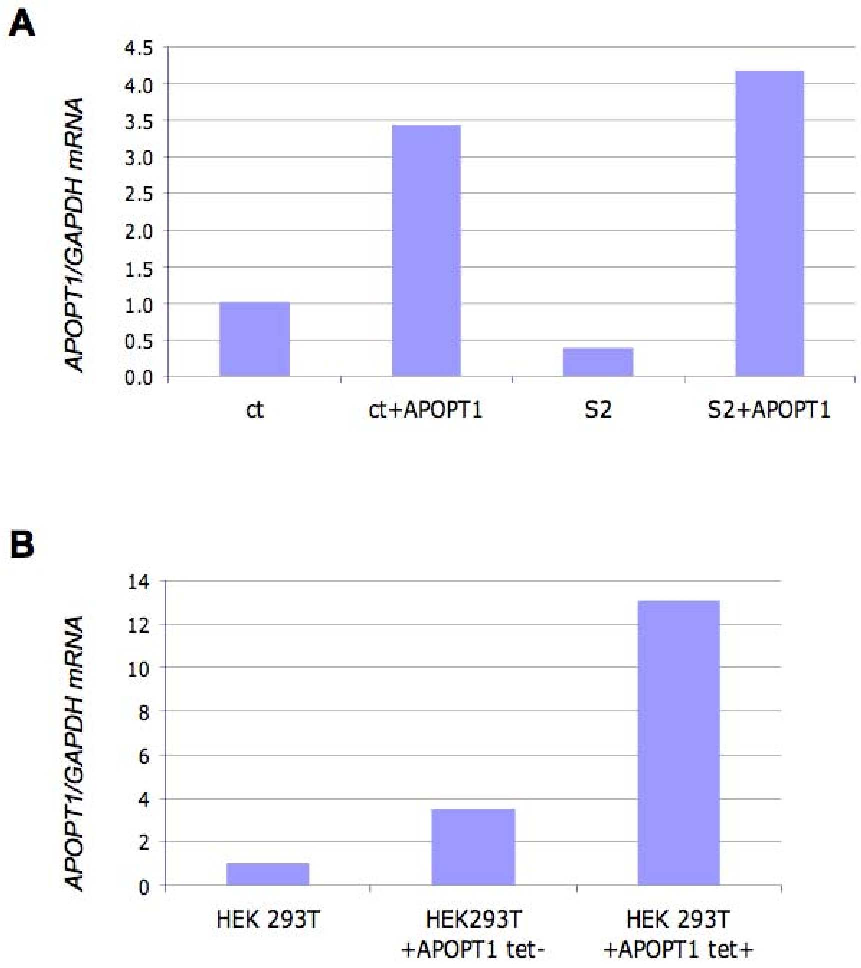

Figure S7

Figure S7. Expression of *APOPT1* in transduced/transfected cell lines

Panel A. Quantitative PCR analysis of *APOPT1* transcript in immortalized fibroblasts from subject 2 (S2) and a control individual (Ct), in basal conditions or stably transduced with *APOPT1-HA* (+APOPT1). The values of *APOPT1* transcript were normalized to *GAPDH*.

Panel B. Quantitative PCR analysis of *APOPT1* transcript in untreated HEK293T cells or in cells transfected with a Tet on-off inducible vector, expressing the *APOPT1-HA* transcript (+APOPT1), grown either without (tet-) or with 1µg/ml doxycycline (tet+).

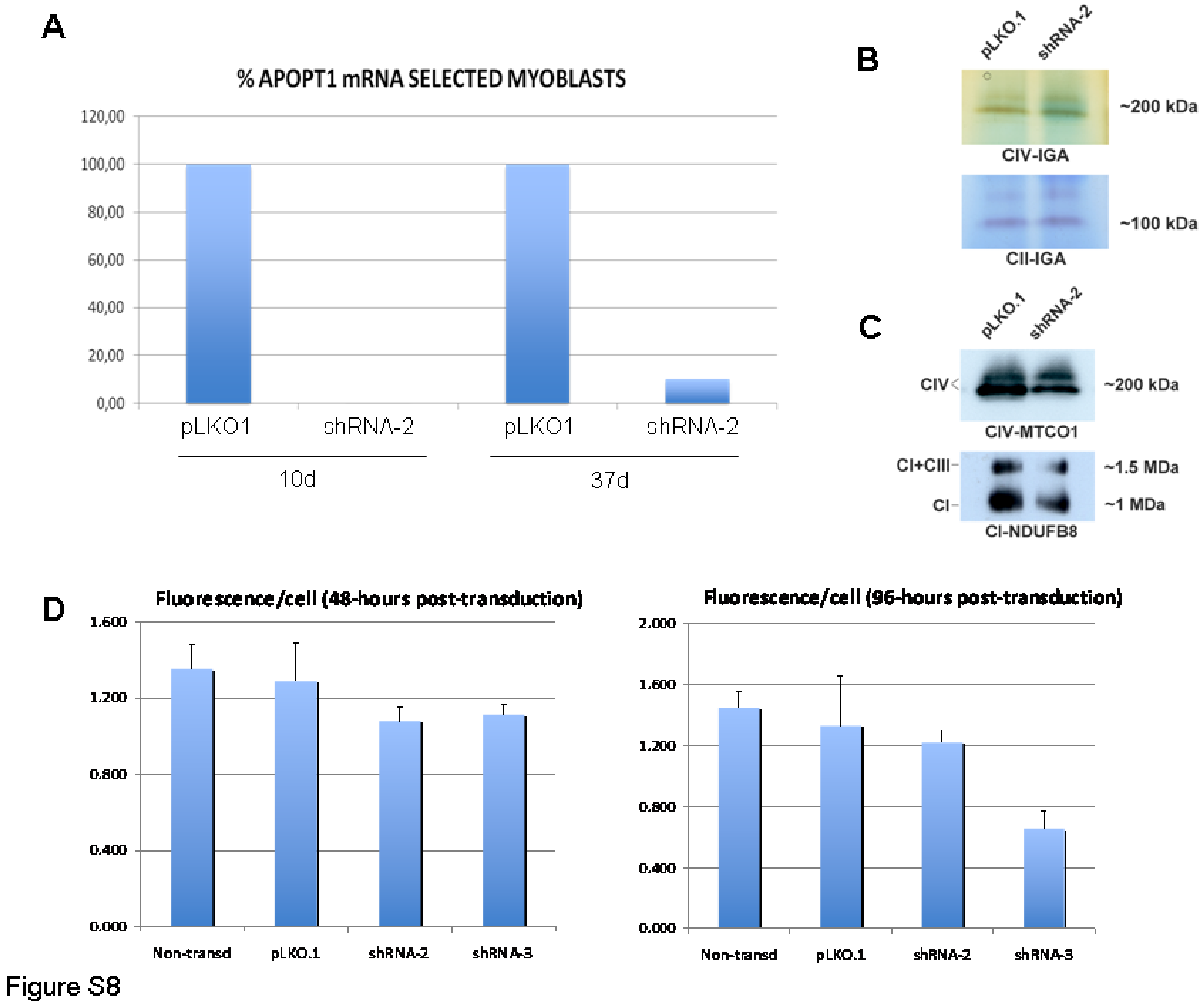

Figure S8. shRNA knockdown of *APOPT1* mRNA

Panel A. Quantitative PCR analysis of *APOPT1* transcript in myoblasts transduced with the "empty" vector pLKO.1, and with *APOPT1* specific shRNA-2. 10d: RNA extracted 10 days post-infection; 37d: 37 days post-infection.

Panel B. In-gel Activity (IGA) for complex IV (CIV) and II (CII) in myoblasts transduced with the "empty" vector pLKO.1 and with *APOPT1* specific shRNA-2. Samples were taken at 37 days post-infection and solubilized with 1% dodecylmaltoside.

Panel C. Western-blot analysis of one-dimension BNGE in myoblasts transduced with the "empty" vector pLKO.1 and with *APOPT1* specific shRNA-2. Samples were taken at 37 days post-infection and solubilized with 1% dodecylmaltoside. We used an antibody against MTCO1 for complex IV (CIV) and an antibody against NDUFB8 to detect complex I (I).

Panel D. ROS detection by dichlorofluorescein (DCHF, Invitrogen) fluorescence on naïve, non-transduced immortalized fibroblasts, fibroblasts transduced with the "empty" vector pLKO.1, and with *APOPT1* specific shRNA-2 and shRNA-3, under exposure to 100uM H_2O_2. Bars represent standard deviations.

Table S1 MRI findings

Subject	S1	S2	S3	S4	S5	S6
Age early MRI (years)	no early MRI available	no early MRI available	4	5	5	3
Cerebral WM abn. - frontal<br>- parietal<br>- occipital<br>- temporal			small lesions in frontal and temporal WM, severe abn. parieto-occipital WM	lesions in R frontal periventricular WM, severe abn. parieto-occipital WM	tiny lesions in frontal and temporal WM, severe abn. parieto-occipital WM	small lesions in frontal and temporal WM, severe abn. parieto-occipital WM
Predominant location WM abn.<br>- lobe<br>- zone			parieto-occipital, periventricular and deep WM	parieto-occipital, periventricular and deep WM	parieto-occipital, periventricular and deep WM	parieto-occipital, periventricular and deep WM
Cysts in abnormal WM			countless	countless	countless	countless
Corpus callosum abn.- genu<br>- body<br>- splenium			entire corpus callosum affected, posterior part most severely	posterior half of corpus callosum severely affected, anterior half spared	tiny lesion in genu; splenium and posterior part body severely affected	one lesion in genu, splenium severely affected, middle segment spared
Internal capsule abn. - anterior<br>- posterior			only posterior limb	only posterior limb	only posterior limb	only posterior limb
Aspect WM abnormalities			inhomogeneous, confluent and multifocal	inhomogeneous, confluent and multifocal	inhomogeneous, confluent and multifocal	inhomogeneous, confluent
Abn. cerebral cortex, basal nuclei, thalami			-	-	-	-
Enlargement lateral ventricles			mild	-	-	-
Enlargement subarachnoid spaces			-	-	-	-
Abn. cerebellar WM and peduncles			-	-	-	-
Abn. cerebellar cortex, dentate nucleus			-	-	-	-
Cerebellar atrophy			-	-	-	-
Abn. midbrain			-	-	parieto-occipito-pontine tracts	parieto-occipito-pontine tracts
Abn. pons			-	-	-	-
Abn. Medulla			-	pyramids	-	-
Contrast enhancement			in multiple foci of abnormal WM	in multiple foci of abnormal WM	in multifocal areas of abnormal WM	no evident enhancement
Restricted diffusion			not investigated	in multifocal areas of abnormal WM	in multifocal areas of abnormal WM	in multifocal areas of abnormal WM
MRS lactate			highly elevated in abnormal WM	highly elevated in abnormal WM	not investigated	highly elevated in abnormal WM

Subject	S1	S2	S3	S4	S5	S6
Age at follow-up MRI (years)	**21**	**15**	**10**	9½	no follow-up MRI	no follow-up MRI
Cerebral WM abn. - frontal<br>- parietal<br>- occipital<br>- temporal	all cerebral WM highly atrophic and abn. signal throughout	only signal abn. in parieto-occipital WM	abn. signal throughout, but most severe in parieto-occipital WM	lesion in R frontal periventricular WM, severe abn. parieto-occipital WM		
Predominant location WM abn.<br>- lobe<br>- zone	no predominance	parieto-occipital, periventricular and deep WM	parieto-occipital, periventricular and deep WM	parieto-occipital, periventricular and deep WM		
WM cysts	multiple small cysts	multiple small cysts	countless, but partially collapsed	countless, but partially collapsed		
Corpus callosum abn.- genu<br>- body<br>- splenium	highly atrophic and abn. in signal throughout	only lesion in splenium	affected throughout and highly atrophic	posterior half of corpus callosum affected, anterior half spared		
Internal capsule abn. - anterior<br>- posterior	only posterior limb	-	only posterior limb	only posterior limb		
Aspect WM abnormalities	confluent	confluent	inhomogeneous, confluent	inhomogeneous, confluent, multifocal		
Abn. cerebral cortex, basal nuclei, thalami	-	-	-	-		
Enlargement lateral ventricles	severe	-	mild	mild		
Enlargement subarachnoid spaces	mild	-	slight	-		
Abn. cerebellar WM and peduncles	subcortical cerebellar WM	-	-	-		
Abn. cerebellar cortex, dentate nucleus	-	-	-	-		
Cerebellar atrophy	+	-	-	-		
Abn. midbrain	pyramidal tracts	-	pyramidal and parieto-occipito-pontine tracts	pyramidal and parieto-occipito-pontine tracts		
Abn. pons	-	-	-	-		
Abn. Medulla	-	-	-	pyramids		
Contrast enhancement	not investigated	not investigated	not investigated	not investigated		
Restricted diffusion	not investigated	not investigated	not investigated	-		
MRS lactate	elevated	not elevated	elevated	not elevated		
Change over time			stabilization and atrophy of abn. areas, collapse of cysts, no new abn.	stabilization and atrophy of abn. areas, collapse of cysts, no new abn., normalization of lactate		

WM, white matter; abn., abnormal/abnormality/abnormalities; -, absent
